# Interspecies variation of larval locomotion kinematics in the genus *Drosophila* and its relation to habitat temperature

**DOI:** 10.1186/s12915-021-01110-4

**Published:** 2021-09-02

**Authors:** Yuji Matsuo, Akinao Nose, Hiroshi Kohsaka

**Affiliations:** 1 Department of Complexity Science and Engineering, Graduate School of Frontier Science, The University of Tokyo, 5-1-5 Kashiwanoha, Kashiwa, Chiba, 277-8561 Japan; 2 Department of Physics, Graduate School of Science, The University of Tokyo, 7-3-1 Hongo, Bunkyo-ku, Tokyo, 133-0033 Japan; 3 School of Informatics and Engineering, The University of Electro-Communications, 1-5-1, Chofugaoka, Chofu-shi, Tokyo, 182-8585 Japan

**Keywords:** Animal locomotion, Larval crawling, The genus Drosophila, Kinematics, Evolution

## Abstract

**Background:**

Speed and trajectory of locomotion are the characteristic traits of individual species. Locomotion kinematics may have been shaped during evolution towards increased survival in the habitats of each species. Although kinematics of locomotion is thought to be influenced by habitats, the quantitative relation between the kinematics and environmental factors has not been fully revealed. Here, we performed comparative analyses of larval locomotion in 11 *Drosophila* species.

**Results:**

We found that larval locomotion kinematics are divergent among the species. The diversity is not correlated to the body length but is correlated instead to the habitat temperature of the species. Phylogenetic analyses using Bayesian inference suggest that the evolutionary rate of the kinematics is diverse among phylogenetic tree branches.

**Conclusions:**

The results of this study imply that the kinematics of larval locomotion has diverged in the evolutionary history of the genus *Drosophila* and evolved under the effects of the ambient temperature of habitats.

**Supplementary Information:**

The online version contains supplementary material available at 10.1186/s12915-021-01110-4.

## Background

Kinematics of animal locomotion is a critical trait enabling each species to survive in their habitats [1]. Movement patterns have been sculpted during their evolution by adaptation to their environments and could have diverged among species [2]. Comparative analyses have identified several examples of differences in the kinematics of locomotion within a group of related species, including insects [3], reptiles [4], birds [5], and primates [6]. To take an example, two gecko species inhabiting either sandy or rocky environments have been shown to exhibit distinct postures [4]. Whereas the interspecies divergence in locomotion patterns can be observed in various phylogenetic branches of the animal kingdom, quantitative comparative analyses of locomotion kinematics remain limited.

Flies of the genus *Drosophila* have long been used as a model to study interspecific diversification and evolution [7]. One salient example of the interspecific variations in the genus *Drosophila* is food for larvae. Some species eat multiple kinds of foods (“generalists” including *Drosophila melanogaster*) while others have strong preferences in food (“specialists” including *Drosophila sechellia*, which has specialised to feed on *Morinda* fruits) [8–10]. These diverged species in the genus *Drosophila* offer an opportunity to perform interspecific comparisons in various animal traits including larval locomotion.

Fly larvae, or maggots, have been widely used in the study of the kinematics of locomotion [11–17]. Among the fly species, *Drosophila melanogaster* (*Dmel*) is one of the most examined species, especially by virtue of the availability of resources in genetics and connectomics [18–21]. *Dmel* larvae locomote by a sequence of forward crawling, and changes of crawling direction are achieved by bending their bodies [11]. The kinematics of larval locomotion is affected by ambient temperature [22–25]. In thermotaxis behaviour in temperature gradient environments, larvae regulate the length of crawling runs between turns and the size and direction of turns [26], and the probability of turns is also affected by ambient temperature gradients [27]. In contrast to the intensive studies on larval behaviour in *Dmel*, locomotion kinematics in the larvae of its sister species in the genus *Drosophila* remains unclear.

Here, we conducted an interspecies comparison of the kinematics of larval locomotion in the genus *Drosophila*. We address two questions in this study: are locomotion kinematics of larvae similar among *Drosophila* species? and if the kinematics are diverged, what factors are related to the diversity? To this aim, we recorded the locomotion of larvae of 11 *Drosophila* species and extracted kinematic parameters using the tracking software FIMTrack [28]. Clustering analysis with Jensen-Shannon divergence and statistical analyses show that two kinematics parameters (bend probability and crawling speed) differ among the *Drosophila* species. We found that kinematics varies with habitat temperature but not with body size. The relationship between the kinematics and minimum habitat temperature is held at two distinct ambient temperatures: 24 °C and 32 °C. Phylogenetic analyses of these kinematics, based on Bayesian inference [29], suggest that the rate of evolution of the kinematics is diverged among phylogenetic branches. Among the eight traits we tested, the evolution of the crawling speed at 24 °C and 32 °C was correlated. Consequently, our results suggest that the kinematics of larval locomotion in the genus *Drosophila* diverged in response to environmental variation in ambient temperature.

## Results

### Kinematic analysis of crawling and bending behaviour in *Drosophila* larvae

**Fig. 1:**
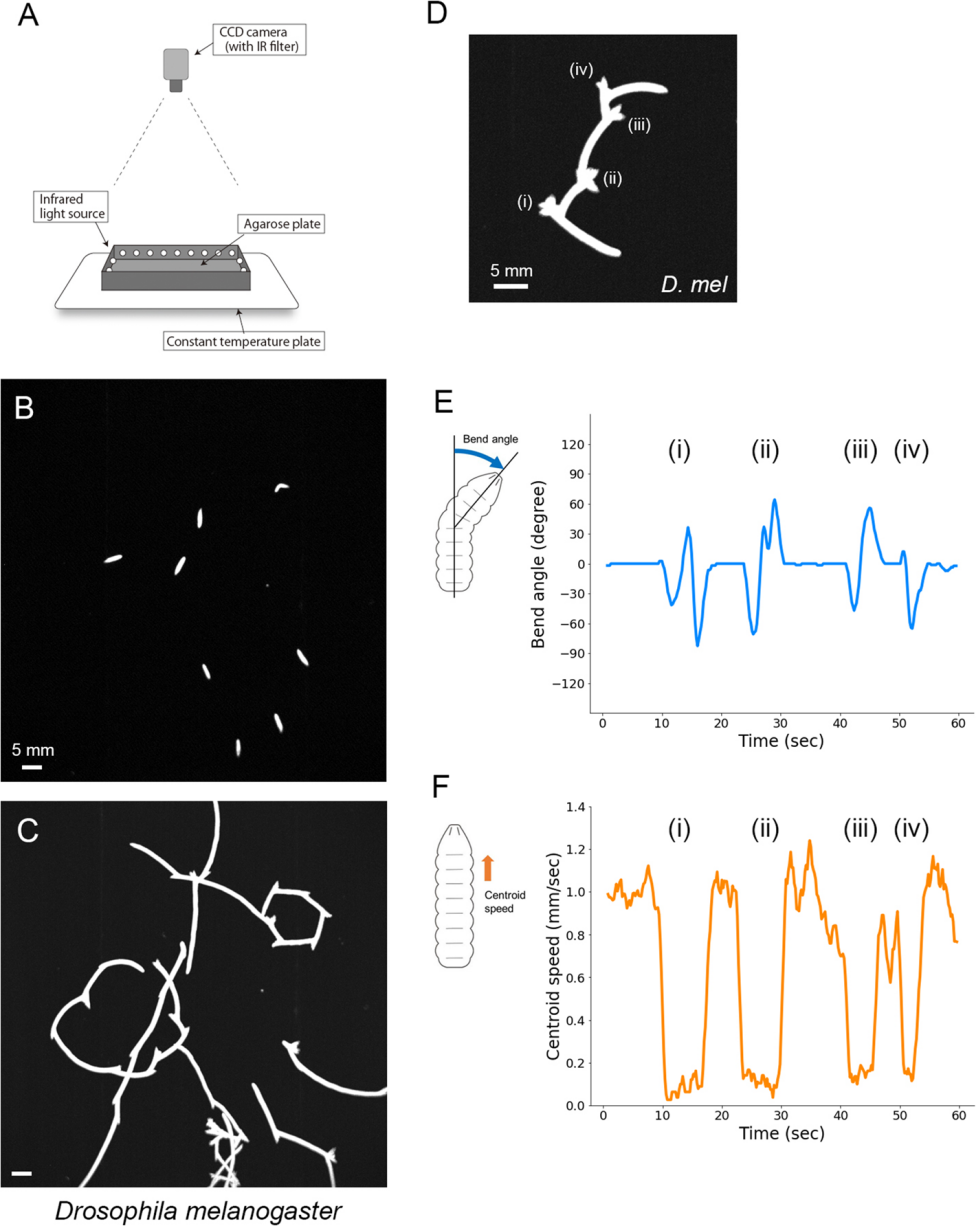
Measurement of larval crawling. **A** Set-up of the recording of larval crawling with infrared light and temperature control plate. **B** An example image of multiple larvae recorded by the set-up **A**. **C** The trajectories of larval locomotion of *Drosophila melanogaster* recorded for 2 min. **D** A trajectory of single *Drosophila melanogaster* locomotion. Locations where the larva changes its direction were labelled (i) to (iv) (**E**, **F**). Bend angle (**E**) and centroid speed (**F**) of the larva in **D**. Labels (i) to (iv) corresponded to those in **D**

In this study, we analysed the kinematics of larval locomotion. To this aim, we recorded the locomotion of fly larvae that were crawling freely on an agarose substrate stage on a temperature-controlled plate (Fig. 1A; see the “Methods” section for details). We placed eight to ten larvae at the centre of the stage, which was kept at 24 °C; illuminated them with infrared light, which was invisible to fly larvae and did not affect their behaviour; and recorded larval locomotion for 3 min at five frames per second (Fig. 1B). Maximum projection of the time-series images showed traces of larval locomotion as multiple curves of multiple larvae (Fig. 1C). In an example of *Drosophila melanogaster* (Fig. 1D), the traces showed smooth curved lines interconnected with angles where larvae exhibited turning behaviour and changed crawling direction, which was consistent with previous studies [11, 30–35]. Larvae under these conditions predominantly exhibited forward locomotion in which muscular contraction was propagating from the posterior to anterior segments. Characteristics of larval locomotion could be described by two measures: the bend angle and centroid speed [31, 33]. The bend angle measures the angle of body axis bending (Fig. 1E), and the centroid speed was the speed of the position of the larval centroid (Fig. 1F). To obtain these values for each larva at each time frame, we used the object tracking software for small animals, FIMTrack [28]. The turning behaviour could be detected by the change in the angle of the body axis (Fig. 1E) and the reduction in the centroid speed (Fig. 1F), as reported previously [31, 33]. Accordingly, we used the bend angle and centroid speed for the quantitative analysis of larval locomotion in this study.

### Classification of locomotion properties in the genus *Drosophila*

**Fig. 2:**
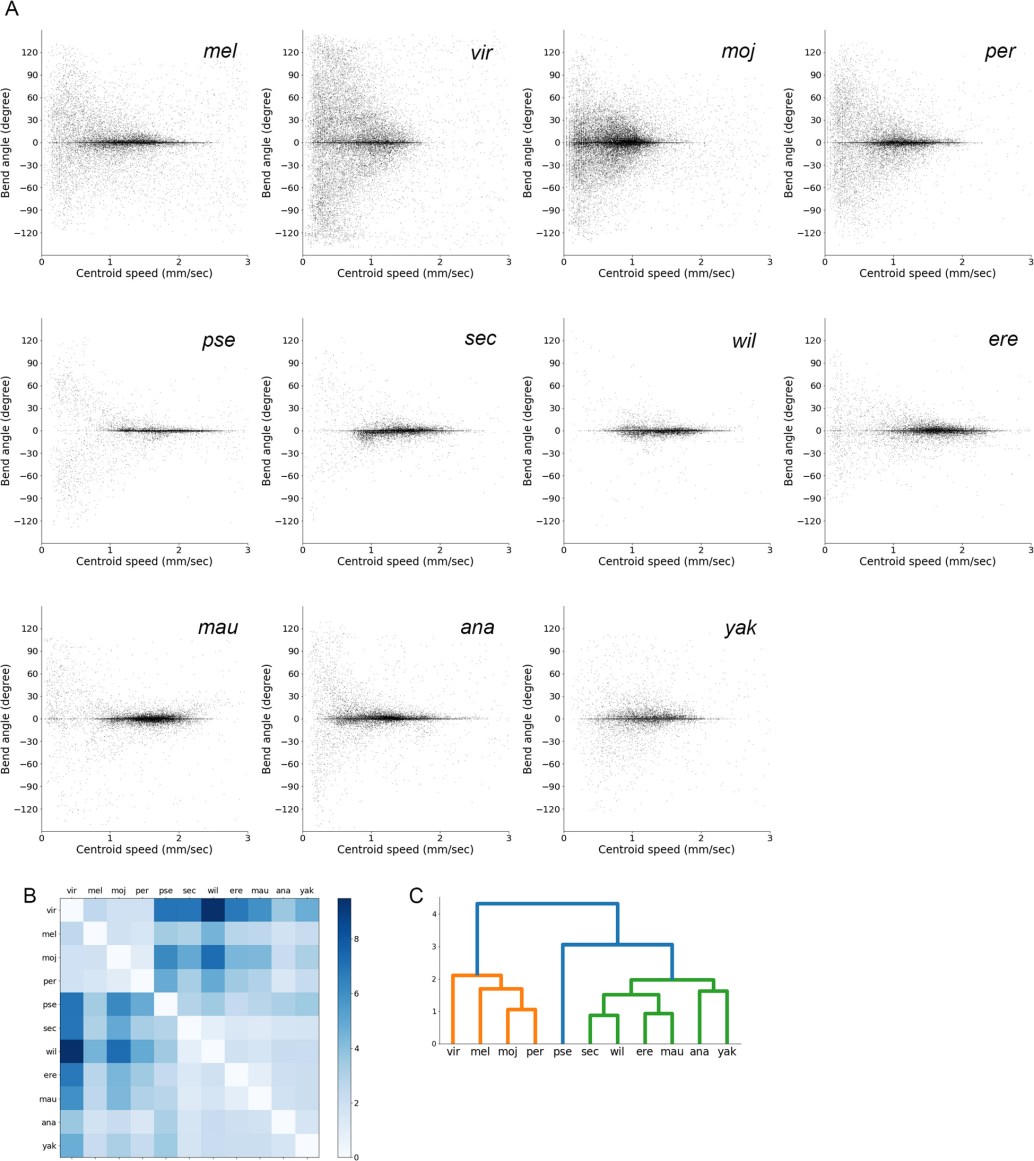
Interspecific comparison of kinematics of larval crawling in the genus *Drosophila*. **A** Plots of the speed of the centroid of larvae and the bend angle in the 11 *Drosophila* species. Each point corresponded to a datum of a single larva of a species in a single time frame. The number of larvae in each species was as follows: *Dvir*: *n* = 24; *Dmel*: *n* = 24; *Dmoj*: *n* = 30; *Dper*: *n* = 27; *Dpse*: *n* = 19; *Dsec*: *n* = 21; *Dwil*: *n* = 18; *Dere*: *n* = 22; *Dmau*: *n* = 26; and *Dana*: *n* = 22; *Dyak*: *n* = 16. **B** Jensen-Shannon divergences of the probability density of **A**. **C** Hierarchical clustering of the kinematics of larval locomotion of the 11 *Drosophila* species

For interspecies comparison of *Drosophila* larval locomotion, we collected 11 fly species in the genus *Drosophila*, whose genome sequences have been read [36, 37] and for which living individuals were available from fly stock centres (KYORIN-Fly, Fly Stocks of Kyorin University; KYOTO Stock Center (DGRC) at the Kyoto Institute of Technology). The 11 *Drosophila* (*D.*, hereafter) species consisted of *D. ananassae* (*Dana*), *D. erecta* (*Dere*), *D. mauritiana* (*Dmau*), *D. melanogaster* (*Dmel*), *D. mojavensis* (*Dmoj*), *D. persimilis* (*Dper*), *D. pseudoobscula* (*Dpse*), *D. sechellia* (*Dsec*), *D. virilis* (*Dvir*), *D. willistoni* (*Dwil*), and *D. yakuba* (*Dyak*). Nine species (all but *Dvir* and *Dmoj*) were classified as subgenus *Sophophora*, and the remaining two (*Dvir* and *Dmoj*) were non-*Sophophora* species. We plotted the centroid speed and bend angle of freely crawling larvae of each species (Fig. 2A). In all the cases, data points accumulated around a region where the bend angle was 0 (Fig. 2A), which reflected an observation that larvae did not bend during the majority of the time (Fig. 1D). We noticed that the deviations from 0 in the bend angle axis were different among species. For example, data points in *Dvir* were scattered along the bend angle axis more than those in *Dwil* (Fig. 2A). To quantify the similarities in the distribution of the two-dimensional plots among the species, we calculated the Jensen-Shannon divergence, which measured the similarity of two probability distributions [38]. We classified the 11 species based on the similarity in the probability distribution of the crawling speed and bend angle plots by hierarchical clustering (Fig. 2B, C). The classification analysis showed that four species (*Dmel*, *Dvir*, *Dmoj*, and *Dper*) form a cluster. These four species showed scattered data points along the bend angle axis compared with the other seven species (Fig. 2A). This observation suggested that the kinematics of larvae in the genus *Drosophila* was diverse among species.

### Definition of the bend probability and crawling speed

**Fig. 3:**
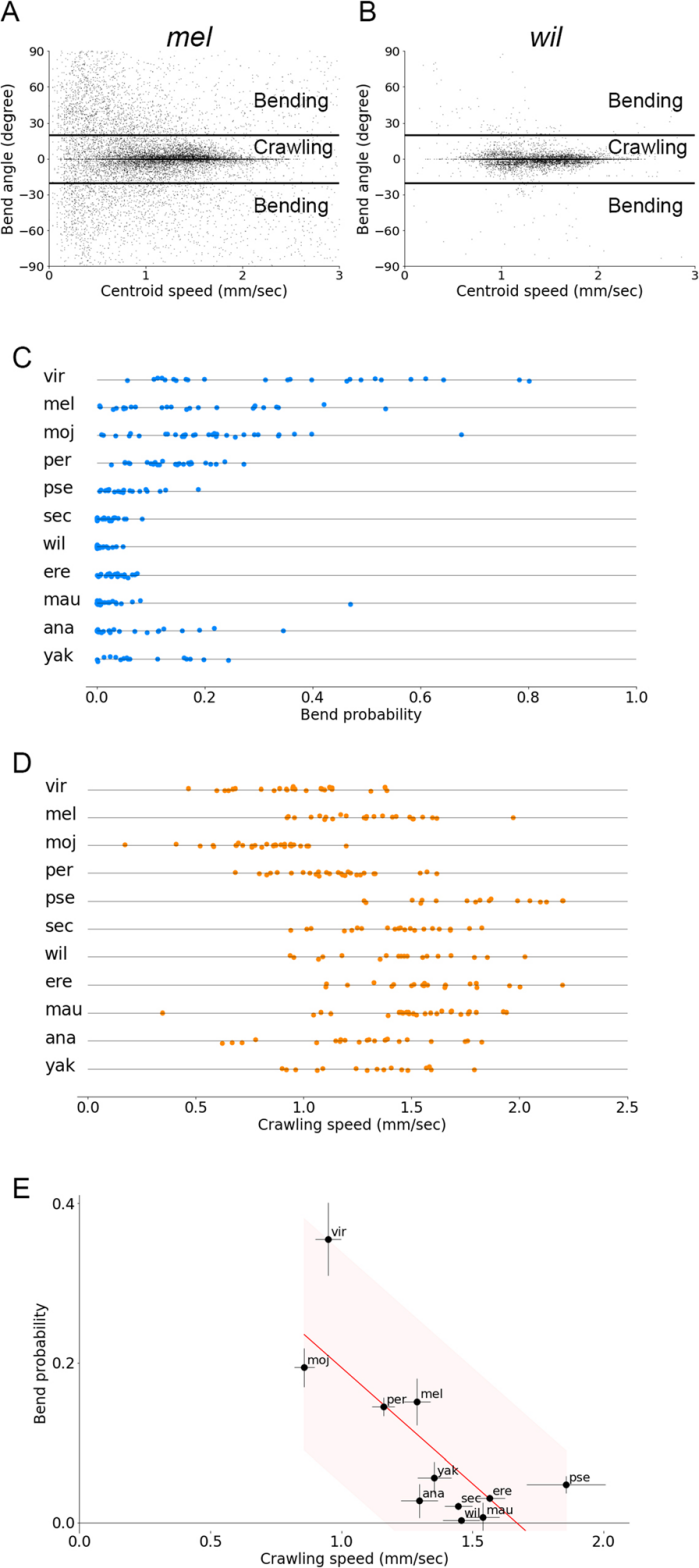
Bend probability and crawling speed of larval locomotion in the 11 *Drosophila* species at 24 °C and their relationship to habitat temperature of each species. **A**, **B** Plots of the speed of the centroid of larvae and the bend angle of *D. melanogaster* (**A**) and *D. willistoni* (**B**). Each point corresponded to a datum of a single larva of a species at a single time frame. Thick horizontal lines denoted the threshold between crawling and bending. **C** Bend probability of individual larvae of each species. **D** Crawling speed of individual larvae of each species. Sample numbers in **C** and **D** were as follows: *Dvir*: *n* = 24; *Dmel*: *n* = 24; *Dmoj*: *n* = 30; *Dper*: *n* = 27; *Dpse*: *n* = 19; *Dsec*: *n* = 21; *Dwil*: *n* = 18; *Dere*: *n* = 22; *Dmau*: *n* = 26; *Dana*: *n* = 22; and *Dyak*: *n* = 16. **E** Scatter plot of bend probability at 24 °C against crawling speed at 24 °C. The median ± sem was shown. The red line showed the linear regression function, and the shaded area represented the 95% confidence band. The point estimate of the Pearson correlation and its 95% confidence interval was − 0.76 and [− .94, − 0.30], respectively

The clustering analysis suggested that the kinematics was divergent among species. To interpret the diversity in terms of locomotion behaviour, we defined two indices: the bend probability and crawling speed. The bend probability measured how often larvae bent their body laterally. We set the minimum angle of the bend as 20°, which was used previously [39], and labelled the larvae that bent more than this threshold angle to the right or left side as “bending” (Fig. 3A, B). This threshold allowed us to extract the difference in the bending rate among the species we examined, and we found that while *Dmel* larvae exhibited larger bend angles than this threshold (Fig. 3A), the bend angles in *Dwil* larvae were mostly less than the threshold (Fig. 3B). We defined the bend probability of every single larva as a ratio of the number of time frames labelled “bending” to the total frame number. To define the second index crawling speed, we labelled the larvae that bent less than the threshold as “crawling” (Fig. 3A, B). We defined the median of centroid speeds of larvae that were labelled “crawling” as the crawling speed for every single larva. By these definitions, we calculated the bend probability and crawling speed of each larva of the species.

### Kinematics of larval locomotion was diverse among the *Drosophila* species

We plotted the bend probability of each species (Fig. 3C). A statistical analysis shows that the bend probability of the species was diverse (*p* = 6.8 × 10^−25^, Kruskal-Wallis test). While *Dvir* larvae exhibited frequent bending (bend probability 0.36 ± 0.05, *n* = 24), *Dwil* larvae rarely bent (bend probability 0.003 ± 0.004, *n* = 18) (Fig. 3C). We also plotted the crawling speed of the *Drosophila* species (Fig. 3D). The statistical analysis showed that the speed was also diverse among the species (*p* = 9.3 × 10^−24^, Kruskal-Wallis test). For example, *Dwil* larvae crawled faster than *Dvir* larvae did (crawling speed 1.46 ± 0.07 mm/s, *n* = 24 in *Dwil*; crawling speed 0.95 ± 0.05 mm/s, *n* = 18 in *Dvir*). These analyses indicated that the bend probability and crawling speed were differentiated in the genus *Drosophila*.

To capture the trends in the diversity of larval kinematics, we plotted the data in the space of the crawling speed and bend probability (Fig. 3E). The graph showed a negative correlation between them (Pearson correlation = − 0.76, *p* = 0.0064). While we used 20° as the threshold for defining the bend probability (Fig. 3A, B), the negative correlation between the crawling speed and bend probability was robust to the choice of the threshold (Pearson correlation = − 0.80, *p* = 0.0030, when the threshold was 10 degrees; Pearson correlation = − 0.71, *p* = 0.014, when the threshold was 30°; Additional file 1: Supplementary Figure 1A - 1C). Furthermore, the median of absolute values of bend angle instead of the bend probability exhibited a negative correlation to the crawling speed (Pearson correlation = − 0.66, *p* = 0.026; Additional file 1: Supplementary Figure 1D). To sum, the crawling speed and bend probability were diverse among the *Drosophila* species and negatively correlated.

### Comparison between intraspecies and interspecies variability

**Fig. 4:**
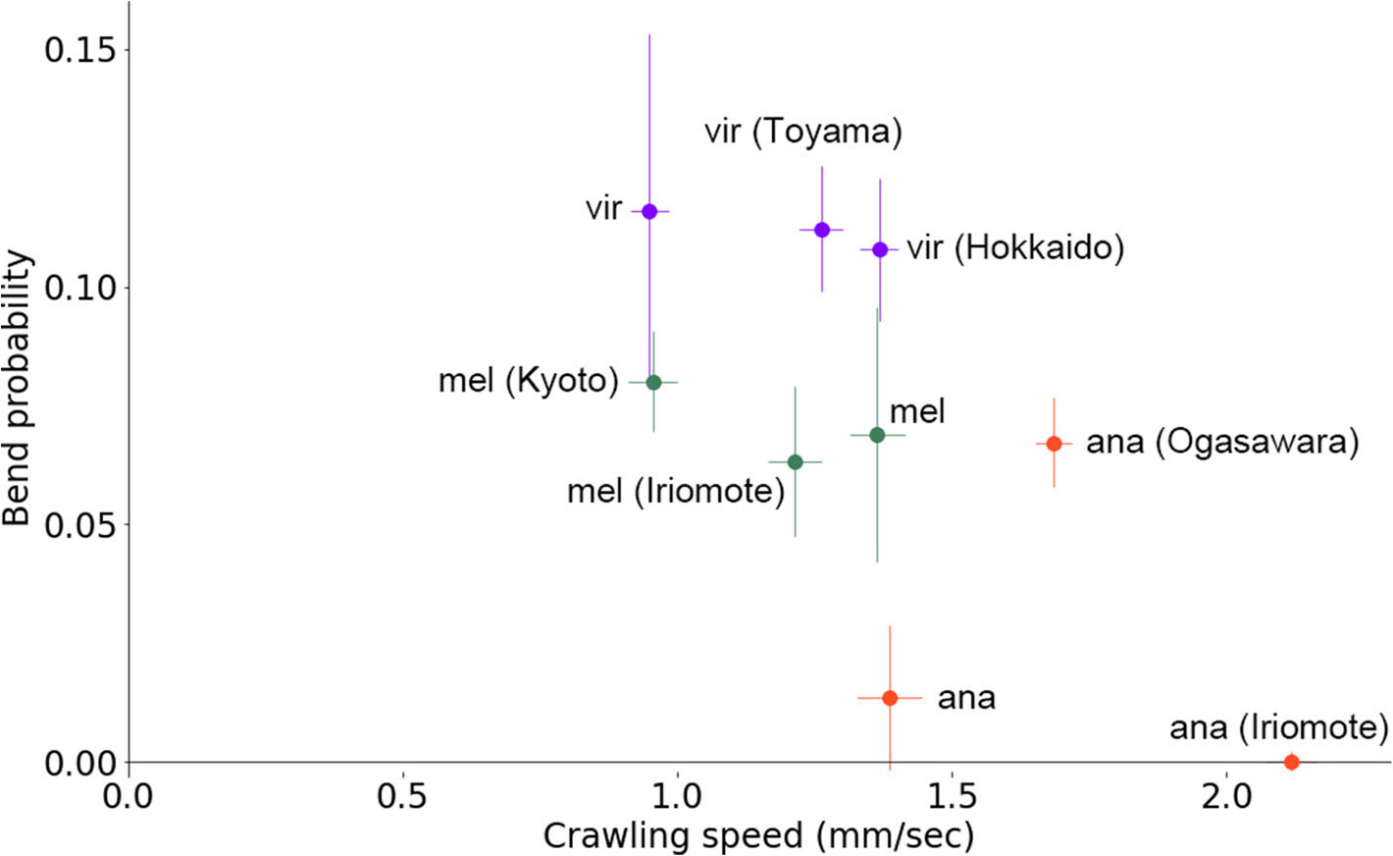
Intraspecific comparison in larval locomotion. Scatter plot of bend probability at 24 °C against crawling speed at 24 °C of nine strains from three species. Sample numbers were as follows: *Dvir*: *n* = 44; *Dvir* (Hokkaido): *n* = 32; *Dvir* (Toyama): *n* = 35; *Dmel*: *n* = 33; *Dmel (Kyoto)*: *n* = 47; *Dmel (Iriomote)*: *n* = 33; *Dana*: *n* = 33; *Dana (Ogasawara)*: *n* = 40; and *Dana (Iriomote)*: *n* = 20

The comparison of the 11 *Drosophila* species represented the natural variability in larval locomotion across them. Here, it should be noted that we used a single strain for each species in the analysis. For that, the variability appeared above could result not only from interspecies but also intraspecies diversity. Even if there was no interspecies variability, sampling data from a population with high intraspecies variation could lead to an apparent diversity across the species. To address this issue, we compared intraspecific deviation with interspecific variability. If intraspecific diversity was a dominant factor for the variability, the deviation within species should be comparable to that among species. In contrast, if interspecific diversity was a major cause, the deviation within species would be smaller than that across species. We examined intraspecies variation in two species in subgenus *Sophophora* (*Dmel* and *Dana*) and one non-*Sophophora* species (*Dvir*) (Fig. 4). Two isofemale strains of each species were obtained from the Kyorin Stock Centre, and the larval kinematics of them were measured. To evaluate the contribution of the interspecific deviations to the total deviations, we conducted the analysis of variance (ANOVA). We found a significant interspecific difference in the bend probability (*p* = 0.008, the one-way ANOVA) although the difference in the crawling speed was marginal (*p* = 0.08, the one-way ANOVA). This observation implied the existence of interspecies diversity in larval locomotion.

### Crawling distance was related to crawling speed and bend probability

**Fig. 5:**
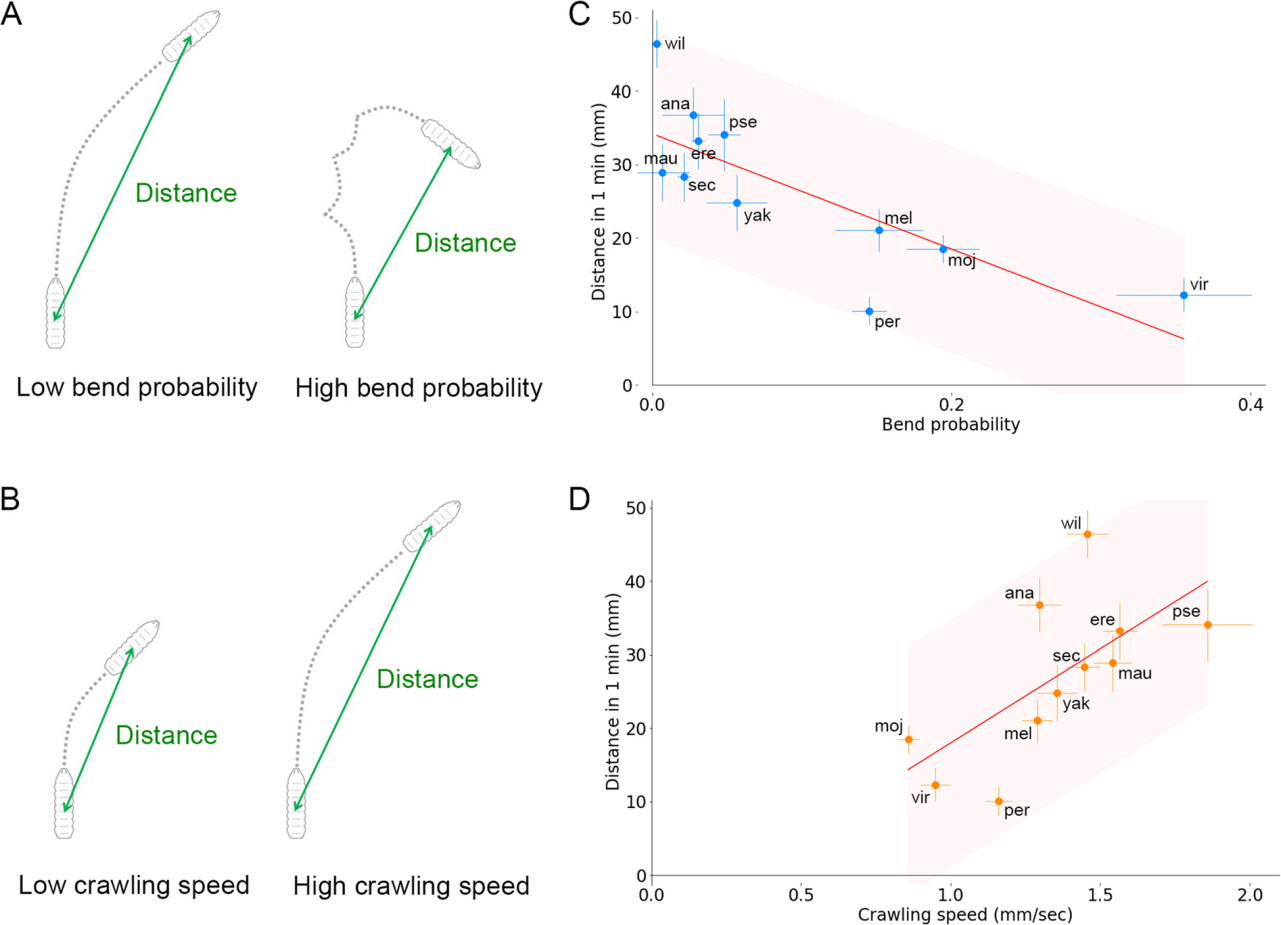
Relationship between crawling distance and the two kinematic parameters in the 11 *Drosophila* species. **A** Schematics of the relationship between the bend probability and crawling distance. **B** Schematics of the relationship between the crawling speed and crawling distance. **C** Scatter plot of the bend probability and crawling distance in 1 min in the 11 species. **D** Scatter plot of the crawling speed and crawling distance in 1 min in the 11 species. The source locomotion data were the same as in Fig. 3C, D. In **C** and **D**, the red lines showed the linear regression functions, and the shaded areas represented the 95% confidence bands. The point estimates of the Pearson correlation and their 95% confidence intervals were − 0.78 and [− 0.94, − 0.34] in **C** and 0.67 and [0.11, 0.91] in **D**, respectively

In the interspecies variability, there was a negative correlation between the crawling speed and bend probability (Fig. 3E). We noticed that this correlation could reflect the control of the crawling distance by a coordinated change of the crawling speed along with the bend probability. The bend probability was negatively correlated with the crawling distance since frequent bending shortened the distance larvae progress in one direction (Fig. 5A) whereas it was obvious that the crawling speed was positively correlated with the crawling distance (Fig. 5B). Accordingly, high crawling speed and low bend probability, which were a combination that appeared in the negative correlation of them (Fig. 3E), both contributed to the increase in the crawling distance. To check whether these geometrical speculations held in the locomotion of larvae, we examined the relationship between the kinematic parameters and the crawling distance (Fig. 5C, D). Consistent with the conjecture, the crawling distance was larger when either the bend probability was lower (Fig. 5C; Pearson correlation = − 0.78) or the crawling speed was higher (Fig. 5D; Pearson correlation = 0.67). These observations implied that the coordinated changes in the crawling speed and bend probability would be related to the change in the crawling distance of larvae of each species.

### No relationship between the kinematics of larval locomotion and the body length nor phylogenetic relationship

Next, we tried to find factors that relate to the diversification of larval kinematics among species. Among the factors in the morphological differences and the ecological diversity that might be involved in the kinematics diversity [3], we examined the body length of larvae as a morphological factor and habitat temperature as an ecological factor. A previous study reported an allometric relationship between the body size and the crawling speed in *Diptera* larvae [13]. The authors analysed larvae in the order *Diptera*; the average size of which spans from 3.7 mm (*Dmel*) to 15.9 mm (*Sarcophaga bullata*). We tested whether the relationship between the body length and the crawling speed also held within the genus *Drosophila*, a subgroup of the order *Diptera*. The length of the *Drosophila* larvae we used spanned from 3.49 ± 0.05 mm (*Dyak*) to 5.68 ± 0.16 mm (*Dpse*) (Additional file 1: Supplementary Figure 2A). We found no significant relationship between the body length and crawling speed (Additional file 1: Supplementary Figure 2B; Pearson correlation = 0.04, *p* = 0.91) and between the body length and the bend probability (Additional file 1: Supplementary Figure 2C; Pearson correlation = 0.55, *p* = 0.077). These data suggested that larval length was not a significant factor for larval kinematics variation within the genus *Drosophila*.

The phylogenetic relationship could also be a factor that affects the kinematics. To test this issue, we focused on two species groups: the obscura species group and the replete species group. *Dper* and *Dpse* belonged to the obscura species group (see Fig. 11A). The kinematics of these two sister species were separated in the distribution of the crawling speed and bend probability (Fig. 3E). Similarly, two species of the replete species group, *Dvir* and *Dmor*, exhibited distinct kinematics (Fig. 3E). These observations suggested that the phylogenetic relationship was not a major factor in the divergence of the kinematics.

### Relationship between the kinematics of larval locomotion and habitat temperature of the *Drosophila* species

**Fig. 6:**
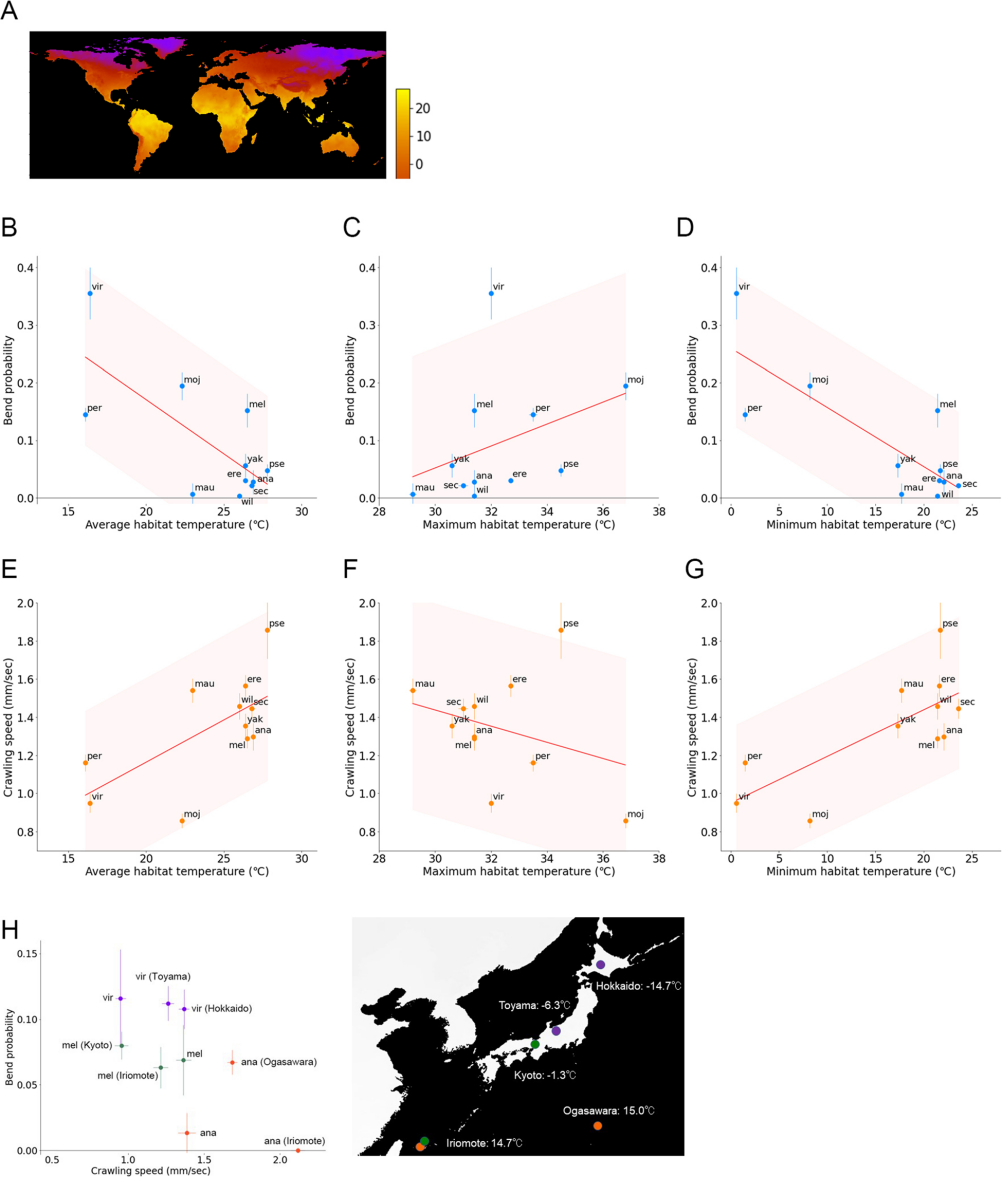
Relationship between the kinematics of larval locomotion and habitat temperature of the *Drosophila* species. **A** A world map of minimum habitat temperature. **B**–**D** Scatter plot of the bend probability of the 11 species at 24 °C against the average (**B**), maximum (**C**), and minimum (**D**) habitat temperatures. **E**–**G** Scatter plot of the crawling speed of the 11 species at 24 °C against the average (**E**), maximum (**F**), and minimum (**G**) habitat temperatures. In **B**–**G**, the median ± sem was shown, and the source locomotion data are the same as in Fig. 3C, D. The red lines showed the linear regression functions, and the shaded areas represented the 95% confidence bands. The point estimates of the Pearson correlation and their 95% confidence intervals were − 0.73 and [− 0.93, − 0.23] in **B**, 0.37 and [− 0.30, 0.79] in **C**, and − 0.81 and [− 0.95, − 0.41] in **D**, 0.66 and [0.09, 0.90] in **E**, − 0.31 and [− 0.77, 0.36] in **F**, and 0.74 and [0.25, 0.93] in **G**. **H** Left: a scatter plot of intraspecific comparison (the same as Fig. 4); right: a map of Japan representing the minimum habitat temperatures of the six strains shown in the left panel

Habitat temperature was one of the critical factors influencing species traits [40]. To test the possible roles of habitat temperature in the evolution of *Drosophila* larval locomotion, we examined the relationship between habitat temperatures and the locomotion kinematics in the genus *Drosophila*. The habitat regions of the 11 species were obtained from the literature [7, 41, 42], and the climate temperature data were obtained from a global climate dataset, WORLDCLIM [43] (Fig. 6A, Additional file 1: Supplementary Figure 3). We examined the relation between larval kinematics (bend probability or crawling speed) and indices of habitat temperatures (Fig. 6B, C). We used the mode (the most frequent value) of three indices of habitat temperatures: average, maximum, and minimum temperature. In the minimum habitat temperature data of *Dvir* and *Dpse*, we noticed that the mode temperatures were below 0 °C, which cannot be a representative habitat temperature for them (Additional file 1: Supplementary Figure 3). Accordingly, for a milder and more representative index, we used the warmest peaks in the minimum habitat temperature histogram (“Tmin” in Additional file 1: Supplementary Figure 3). Considering the uneven distribution of flies within the region demarcated in Additional file 1: Supplementary Figure 3, the temperature of the warmest peak in the minimum habitat temperature might represent the coldest temperature the majority of the population of each species experiences over many years.

We found that the bend probability and crawling speed were both correlated with average habitat temperature Tave (Fig. 6B, E. Pearson correlation: bend probability vs Tave = − 0.73, crawling speed vs Tave = 0.66). We further analysed whether the maximum and minimum temperature contribute to the correlation. Maximum habitat temperature, Tmax, showed no obvious correlation to the kinematics (Fig. 6C, F. Pearson correlation: bend probability vs Tmax = 0.37, crawling speed vs Tmax = − 0.31). In contrast, the minimum habitat temperature, Tmin, exhibited a stronger correlation than Tmax (Fig. 6D, G. Pearson correlation: bend probability vs Tmin = − 0.81, crawling speed vs Tmin = 0.74). Even after omitting extreme values (*Dvir* in the bend probability data and *Dpse* in the crawling speed data), the correlation remained high (Pearson correlation: the bend probability vs Tmin (without *Dvir*) = − 0.69, the crawling speed vs Tmin (without *Dpse*) = 0.78). Furthermore, even considering multiple comparisons (four factors: larval length, Tave, Tmax, and Tmin), the correlations between the kinematic parameters and Tmin were statistically significant (*p* = 0.039 in crawling speed vs Tmin, *p* = 0.0010 in bend probability vs Tmin; Bonferroni correction).

The range (or variability) of habitat temperature could also be a critical factor to determine larval kinematics because species that inhabits in highly variable temperature area would be insensitive to the change in the ambient temperature whereas those breeding in a narrow range of temperature would be sensitive to the small shift in the ambient temperature. To test this point, we examined the relationship between the larval kinematics and the range of habitat temperature and the difference between the maximum and minimum temperature in their habitat area (Additional file 1: Supplementary Figure 4). The range of habitat temperature was correlated with both the bend probability (Pearson correlation = 0.65, *p* = 0.030) and the crawling speed (Pearson correlation = − 0.69, *p* = 0.0019). Consequently, the range of habitat temperature could affect the larval kinematics. In the following analysis, we focused on the minimum temperature Tmin since it showed the most evident correlation to the larval kinematics.

It should be noted that the strains we used have been kept at the housed stocks at 23 or 20 °C (see the “Methods” section), which might affect the innate behaviour in wild-type strains. However, a comparison between the time period after speciation (at least million years ~ 10^7^ generations [44, 45]) and that in laboratories (100 years ~ 10^3.5^ generations) indicated that the duration in laboratories occupied as small as 0.03% of the time for the evolution of these *Drosophila* strains. In addition, the rearing temperatures in the stock centres were within the range of habitat temperature of each species (Additional file 1: Supplementary Figure 3). These rearing conditions implied that the strains we collected from the stock centre should possess innate behaviour that was evolved in their habitat.

To sum, larvae of species inhabiting moderate environments (where Tmin is 15 to 25 °C) showed low bend probability and fast crawling, or long crawling distance, whereas those inhabiting cold environments (where Tmin is 0 to 10 °C) exhibited frequent bending and slow crawling, or short crawling distance. Especially, species that had lower Tmin than the ambient temperature in this assay (24 °C) showed shorter crawling distance, which implied that excess heat stimuli to these species should reduce their crawling distance.

In the analysis above, we estimated the indices of habitat temperature from the temperature data in the entire potential habitat of each species because the original habitat of each strain was unclear. To further check the correlation between the larval kinematics and habitat temperature, we examined the relationship between the kinematics and habitat temperature among the strains of which habitat was recorded (Figs. 4 and 6H). We used two *Dmel* strains (collected at Kyoto and Iriomote in Japan), two *Dvir* strains (collected at Hokkaido and Toyama in Japan), and two *Dana* strains (collected at Ogasawara and Iriomote in Japan). The habitat temperature at these locations for collecting flies was diverse (Fig. 6H, right). Intriguingly, the strains originated from the north part of Japan, where the minimum habitat temperature was low, exhibited slower crawling and high bend probability whereas those from the south part of Japan, where the minimum habitat temperature was high, showed the opposite trend (Fig. 6H, left), which was consistent with the observation based on the temperature of the worldwide statistics (Fig. 6D, G). Consequently, these observations implied that habitat temperature should be one of the leading factors in sculpting the kinematics of larval locomotion in the genus *Drosophila*.

### Correlation between bend probability and habitat temperature among *Drosophila* species at high ambient temperature

**Fig. 7:**
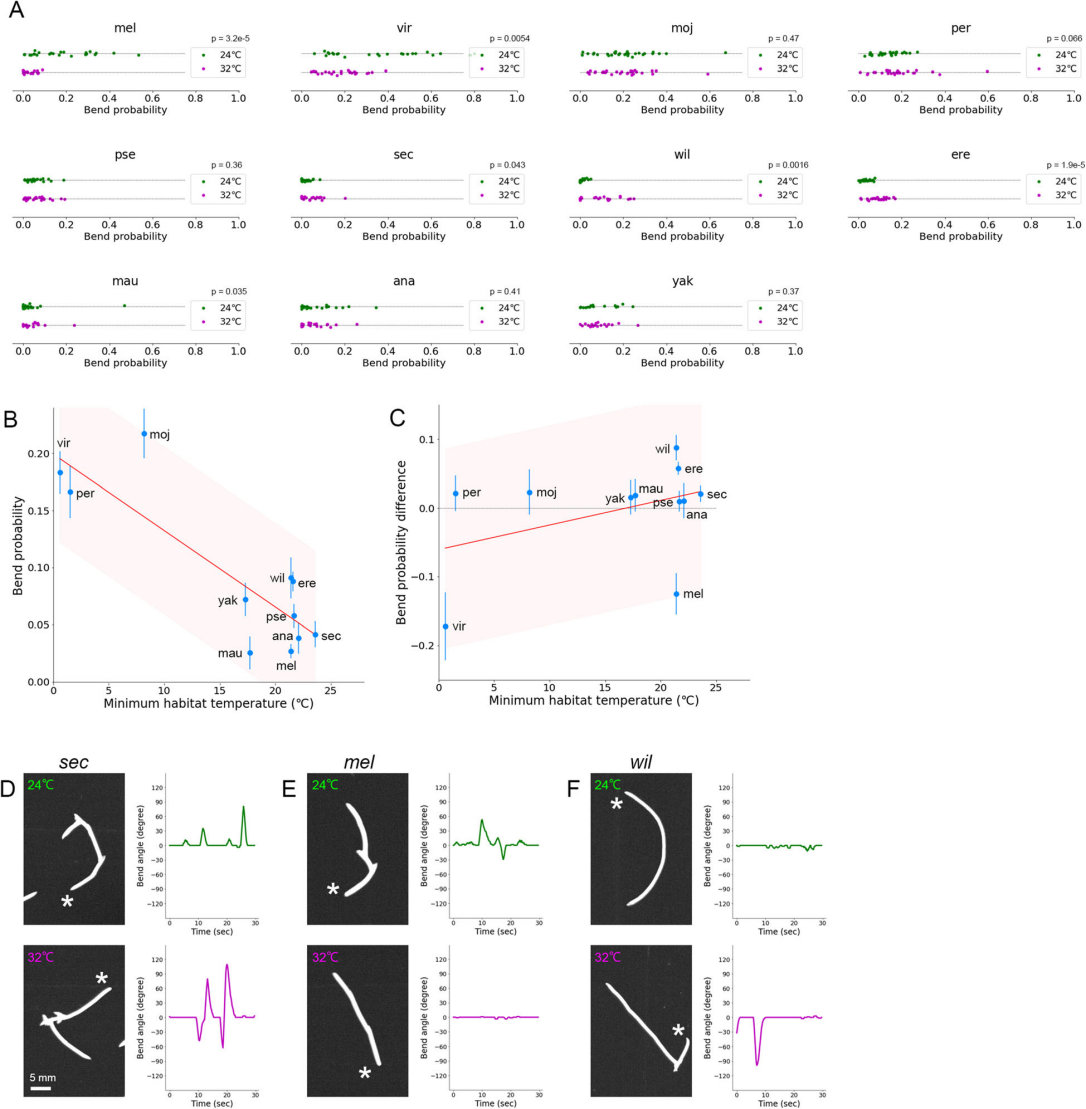
Bend probability in larval locomotion in the 11 *Drosophila* species at 32 °C. **A** Bend probability of the 11 species at 24 °C and 32 °C. The data at 24 °C were the same as in Fig. 3C. Sample numbers at 32 °C were as follows: *Dvir*: *n* = 25; *Dmel*: *n* = 20; *Dmoj*: *n* = 29; *Dper*: *n* = 27; *Dpse*: *n* = 25; *Dsec*: *n* = 20; *Dwil*: *n* = 21; *Dere*: *n* = 24; *Dmau*: *n* = 17; *Dana*: *n* = 23; and *Dyak*: *n* = 19. *p* values presented the results of the Mann-Whitney *U* test. **B** Scatter plot of bend probability at 32 °C against the minimum habitat temperature, Tmin. **C** Scatter plot of the difference in bend probability between 32 and at 24 °C (bend probability at 32 °C − bend probability at 24 °C) against the minimum habitat temperature Tmin. In **B** and **C**, the median ± sem was shown. The red lines showed the linear regression functions, and the shaded areas represented the 95% confidence bands. The point estimates of the Pearson correlation and their 95% confidence intervals were − 0.850 and [− 0.96, − 0.51] in **B** and 0.40 and [− 0.26, 0.81] in **C**, respectively. **D**–**F** Examples of trajectories (left) and the bend angle (right) at 24 °C (top) and 32 °C (bottom) of larval locomotion of each species (**D** *Dsec*, **E** *Dmel*, and **F** *Dwil*)

**Fig. 8:**
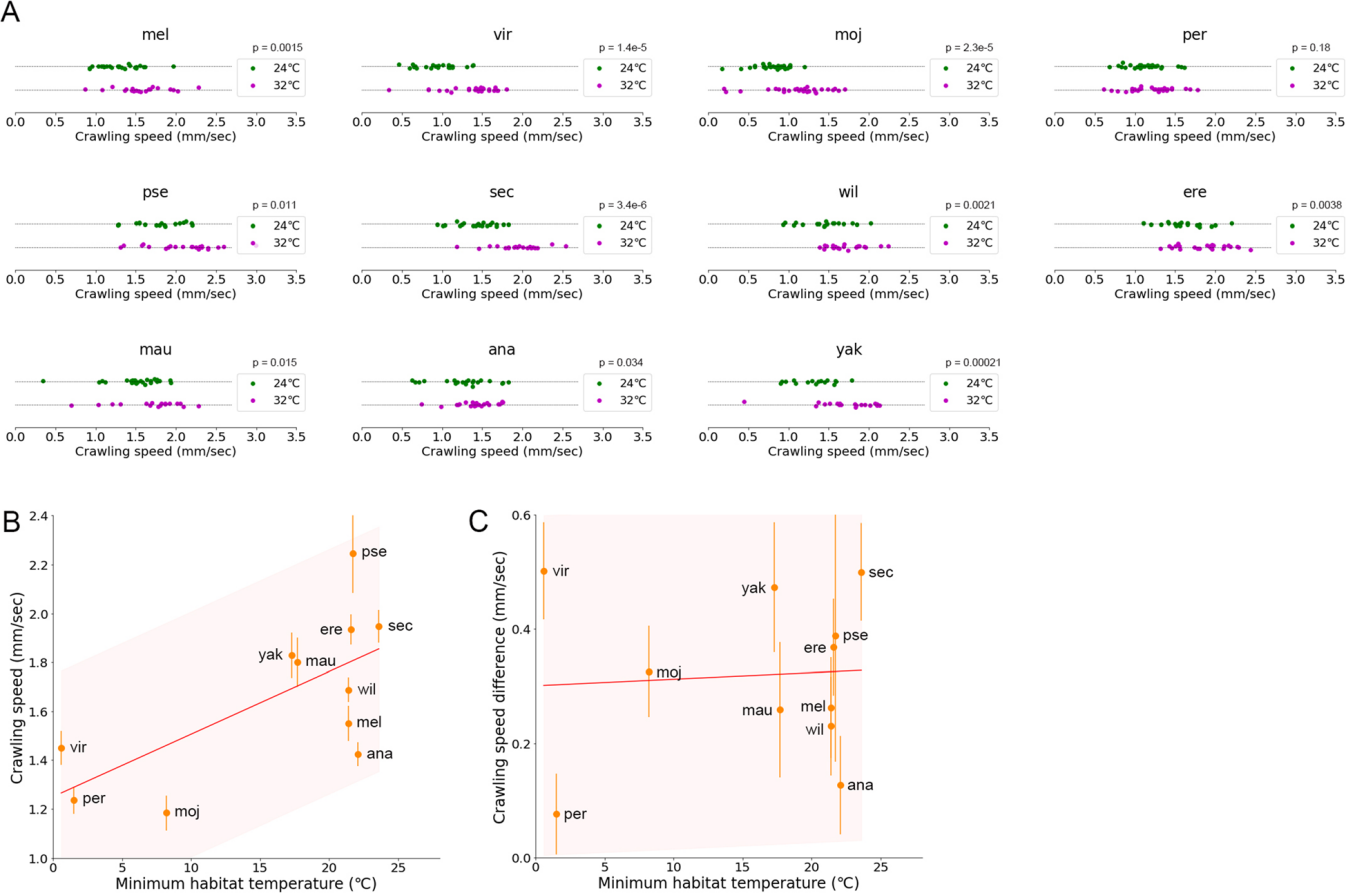
Crawling speed of larval locomotion in the 11 *Drosophila* species at 32 °C. **A** Crawling speed of the 11 species at 24 °C and 32 °C. The data at 24 °C were the same as in Fig. 3D. Sample numbers at 32 °C were as follows: *Dvir*: *n* = 25; *Dmel*: *n* = 20; *Dmoj*: *n* = 29; *Dper*: *n* = 27; *Dpse*: *n* = 25; *Dsec*: *n* = 20; *Dwil*: *n* = 21; *Dere*: *n* = 24; *Dmau*: *n* = 17; *Dana*: *n* = 23; and *Dyak*: *n* = 19. *p* values presented the results of the Mann-Whitney *U* test. **B** Scatter plot of the crawling speed at 32 °C against the minimum habitat temperature Tmin. **C** Scatter plot of the difference of the crawling speed between at 32 and at 24 °C (crawling speed at 32 °C − crawling speed at 24 °C) against the minimum habitat temperature Tmin. In **B** and **C**, the median ± sem was shown. The red lines showed the linear regression functions, and the shaded areas represented the 95% confidence bands. The point estimates of the Pearson correlation and their 95% confidence intervals were 0.67 and [0.12, 0.91] in **B** and 0.07 and [− 0.56, 0.64] in **C**, respectively

So far, we analysed larval locomotion at 24 °C, which was close to the rearing temperature (see the “Methods” section). Larvae of *Dmel* were known to crawl faster at 32 °C than at 25 °C [25]. So, we next examined whether the relation between the kinematics indices and habitat temperature held or not, and how the kinematics indices were changed, at a higher ambient temperature. We performed the same set of measurements and analysis of larval locomotion at 32 °C (Figs. 7 and 8). The interspecies comparison showed that the bend probabilities at 32 °C were correlated with minimum habitat temperature (Fig. 7B; Pearson correlation = − 0.85, *p* = 0.0009). To examine the effects of ambient temperature on the bend probability in detail, we compared the differences of bend probability at 24 °C and 32 °C among the species (Fig. 7C). We found no significant relationship between the change in bend probability and the minimum habitat temperature (Fig. 7C; Pearson correlation = 0.40, *p* = 0.22). Seven species showed no significant changes in the bend probability at 32 °C (Mann-Whitney *U* test of *Dmoj*, *Dper*, *Dpse*, *Dsec*, *Dmau*, *Dana*, and *Dyak* in Fig. 7A–D), while *Dvir* and *Dmel* showed a decrease in the bend probability at 32 °C, and *Dwil* and *Dere* exhibited an increase (Mann-Whitney *U* test in Fig. 7A–C, E, F). Accordingly, we concluded that while the shift of the bend probability between the distinct temperatures was diverse among the *Drosophila* species, the overall trend between the bend probability in relation to habitat temperature held at the higher ambient temperature of 32 °C.

### Correlation between crawling speed and habitat temperature among *Drosophila* species at high temperature

Next, we analysed the crawling speed at 32 °C (Fig. 8A). Similar to the bend probability, we found that the crawling speed at 32 °C was correlated with the minimum habitat temperature among the species, as it was at 24 °C (Fig. 8B; Pearson correlation = 0.67, *p* = 0.024). To examine the effects of ambient temperature on the crawling speed in detail, we compared the difference in the crawling speed at 24 to 32 °C among the species (Fig. 8C). In contrast to the case in the bend probability, shifts in the crawling speed among the species were common: ten species (all the species but *Dper*) showed a significant increase in crawling speed at 32 °C (Mann-Whitney *U* test in Fig. 8A). *Dper* also exhibited an increase in the crawling speed, but it was not statistically significant (Mann-Whitney *U* test in Fig. 8A). Accordingly, at 32 °C, larvae of all the species we tested increased their speed of crawling, which might reflect a general demand to avoid malfunctions in metabolic reactions and dehydration of the body. We also tested the relationship between the change in the speed at 32 °C and the minimum habitat temperature for each species (Fig. 8C). We found no significant correlation between the speed change and the habitat temperature (Fig. 8C; Pearson correlation = 0.07, *p* = 0.84), which also implied that the speeding up at high temperature was a general requirement among the species for larval survival. Accordingly, whereas the details of shifts were different (diverse shift in the bend probability and common shift in the crawling speed among the species), the overall relationship between the kinematic indices and minimum habitat temperature was also held at the higher ambient temperature of 32 °C.

### A kinematic trend of larval locomotion in the genus *Drosophila*

**Fig. 9:**
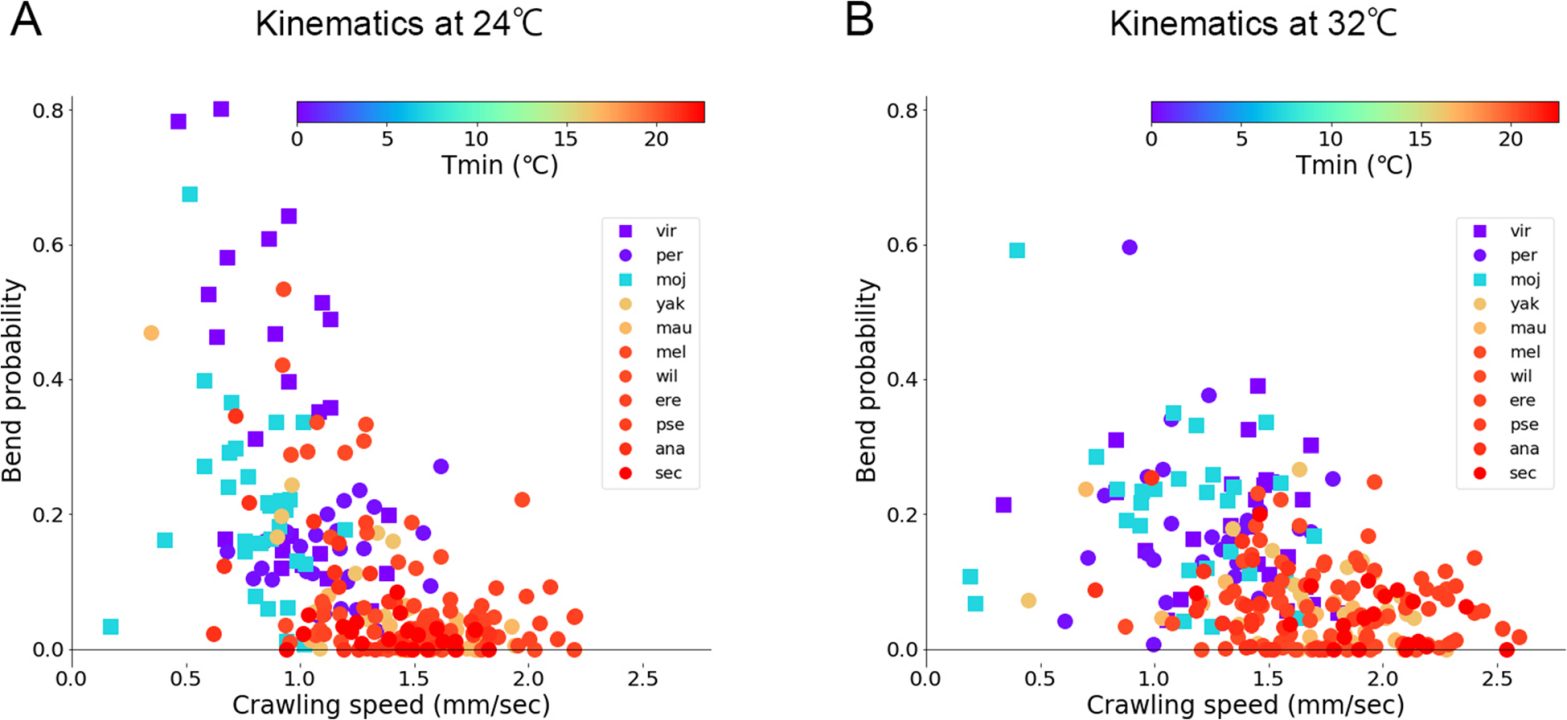
Scatter plot of the kinematics in the 11 species at 24 °C and 32 °C. **A**, **B** Two-dimensional plots of the crawling speed and bend probability. Each point corresponded to the data of a single larva of the species. The colour of markers denoted minimum habitat temperature (Tmin) as shown in the colour bar. Circles denoted *Sophophora* species, and squares denoted non-*Sophophora* species. The source locomotion data were the same as Figs. 3, 7, and 8. **A** 24 °C. **B** 32 °C

To see any gross trend in the shift of larval kinematics in the different species at distinct ambient temperatures, we plotted the kinematics of individual larvae of all the species (Fig. 9). At the ambient temperature of 24 °C, a trend of kinematics was observed in which the data points were distributed from low crawling speed/high bend probability (the top-left corner in the plot) to high crawling speed/low bend probability (the bottom-right corner in the plot) as the minimum habitat temperature increased (Fig. 9A). Intriguingly, the kinematics indices between distinct species were not segregated but rather overlapped. This continuum property can also be observed in the kinematics data at the ambient temperature of 32 °C (Fig. 9B). According to the modern phylogenetic concept [7], the 11 genus *Drosophila* species in our study consisted of nine subgenus *Sophophora* species (all but *Dvir* and *Dmoj*) and two non-*Sophophora* species (*Dvir* and *Dmoj*). In our plots of kinematics, species in the non-*Sophophora* (*Dvir* and *Dmoj*) were located at the left side in the continuum (Fig. 9). This observation implied that the kinematics indices of larvae in *Sophophora* species in the genus *Drosophila* took values in this continuum and kinematics indices in non-*Sophophora* species in the genus *Drosophila* diverged along this continuum by adaptation to habitat temperature during evolution.

### No correlation between crawling speed and habitat temperature among *Drosophila* species at an extreme ambient temperature of 40 °C

**Fig. 10:**
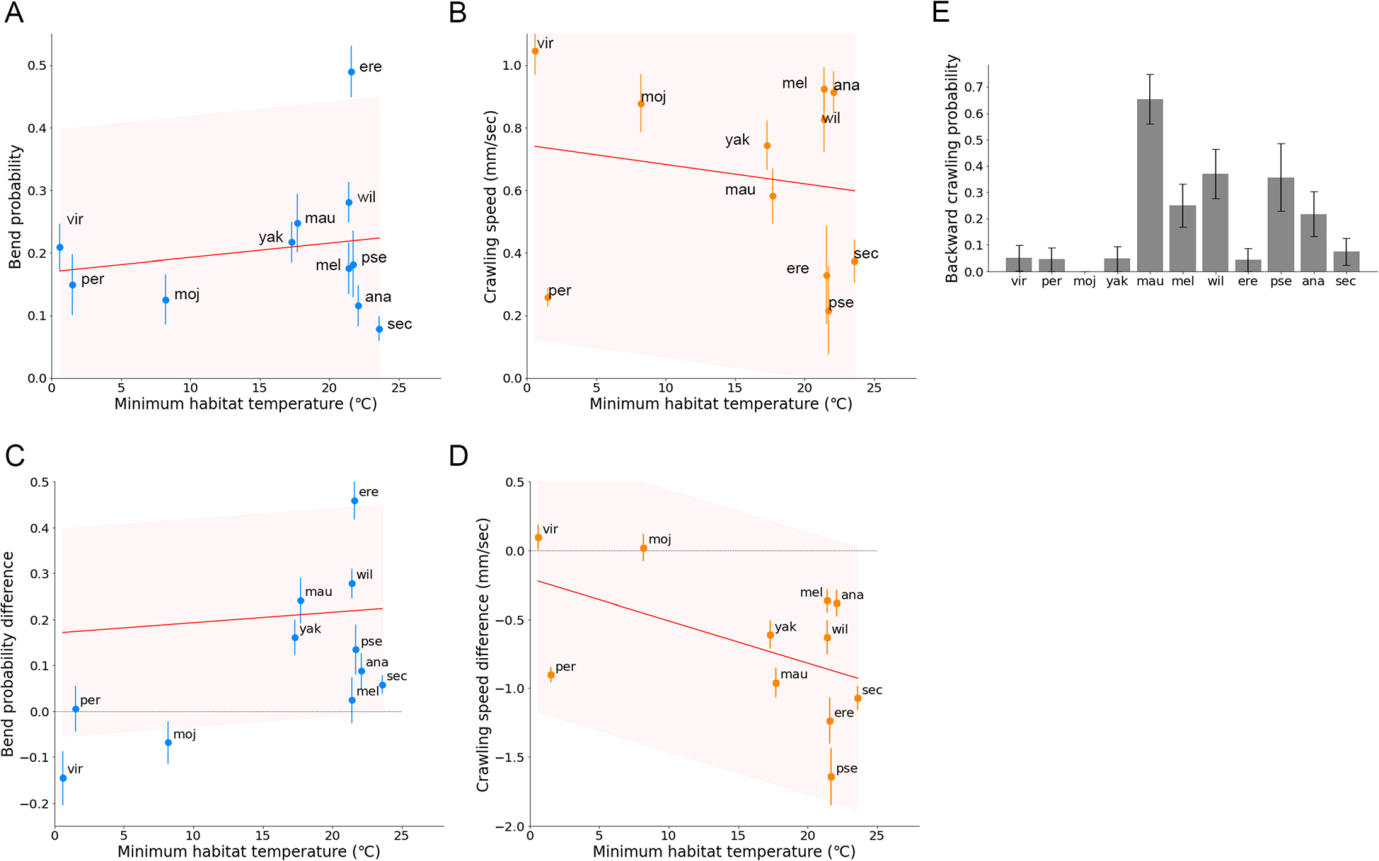
Larval behaviour in the 11 species at 40 °C. **A** Scatter plot of the bend probability at 40 °C against the minimum habitat temperature Tmin. **B** Scatter plot of the crawling speed at 40 °C against the minimum habitat temperature Tmin. **C** Scatter plot of the difference of the bend probability between at 40 and at 24 °C (bend probability at 40 °C − bend probability at 24 °C) against the minimum habitat temperature Tmin. **D** Scatter plot of the difference of the crawling speed between 40 and at 24 °C (crawling speed at 40 °C − crawling speed at 24 °C) against the minimum habitat temperature Tmin. The median ± sem was shown in **A**–**D**. Sample numbers for data at 40 °C were as follows: *Dvir*: *n* = 20; *Dmel*: *n* = 28; *Dmoj*: *n* = 23; *Dper*: *n* = 21; *Dpse*: *n* = 16; *Dsec*: *n* = 27; *Dwil*: *n* = 27; *Dere*: *n* = 22; *Dmau*: *n* = 26; *Dana*: *n* = 23; and *Dyak*: *n* = 20. Sample numbers for data at 24 °C were the same as Fig. 3C, D. The red lines showed the linear regression functions, and the shaded areas represented the 95% confidence bands. The point estimates of the Pearson correlation and their 95% confidence intervals were 0.17 and [− 0.48, 0.70] in **A** and − 0.17, [− 0.70, 0.48] in **B**, 0.17, [− 0.48, 0.70] in **C**, and − 0.50, [− 0.84, 0.15] in **D**. **E** Backward crawling probability at 40 °C of each species. Probability ± standard error based on the binomial distribution was shown. Sample numbers in **E** were as follows: *Dvir*: *n* = 20; *Dmel*: *n* = 28; *Dmoj*: *n* = 23; *Dper*: *n* = 22; *Dpse*: *n* = 14; *Dsec*: *n* = 27; *Dwil*: *n* = 27; *Dere*: *n* = 23; *Dmau*: *n* = 26; *Dana*: *n* = 23; and *Dyak*: *n* = 21

Temperatures of 24 °C and 32 °C were within the range of natural habitat temperatures of most of the species we tested (Additional file 1: Supplementary Figure 3). We next examined larval crawling at an extreme temperature and investigated the relation between the kinematics indices and minimum habitat temperature. Too high temperature could be noxious for many animals, including *Drosophila* larvae. When *D. melanogaster* larvae were stimulated with a probe heated to 42 °C, they exhibited a stereotyped rolling motion as an escape behaviour [46–48]. At below 40 °C, on the other hand, larvae did not exhibit rolling behaviour [47], which allowed us to study crawling kinematics in this semi-noxious extreme environment. We recorded larval locomotion of the *Drosophila* species at the ambient temperature of 40 °C, which corresponded to the highest edge of maximum habitat temperature histograms for many of the species (Additional file 1: Supplementary Figure 3). We measured the bend probability and crawling speed of the 11 *Drosophila* species (Additional file 1: Supplementary Figures 5 and 6). At the ambient temperature of 40 °C, neither the bend probability nor crawling speed was correlated with minimum habitat temperature (Fig. 10A, B; Pearson correlation: bend probability at 40 °C vs Tmin = 0.17, *p* = 0.61; crawling speed at 40 °C vs Tmin = − 0.17, *p* = 0.61). Since 40 °C was close to the maximum habitat temperature rather than the minimum habitat temperature, we analysed the relation between the kinematics indices and maximum habitat temperature instead of the minimum habitat temperature. However, neither the bend probability nor crawling speed was correlated with maximum habitat temperature (Pearson correlation: bend probability at 40 °C vs maximum habitat temperature = − 0.13, *p* = 0.71; crawling speed vs maximum habitat temperature = − 0.10, *p* = 0.77). Accordingly, the relation between the kinematic indices and habitat temperature did not hold at the extreme ambient temperature of 40 °C. This observation suggested that the influence of habitat temperature on the evolution of the locomotion kinematics was restricted within a specific range of ambient temperatures. To examine the effects of the extreme ambient temperature on the kinematics indices in detail, we examined the shift in the bend probability at the distinct temperatures of 40 °C and 32 °C among the species (Additional file 1: Supplementary Figure 5). In seven species, bend probability increased at 40 °C. Three species that inhabited moderate temperature areas (*Dvir*, *Dper*, and *Dmoj*) and one species that lived on an isolated island (*Dsec*) showed little change in the bend probability, which might reflect a distinct strategy in evolution to cope with the semi-noxious environment. We also examined the shift in the crawling speed at the distinct temperatures of 40 °C and 32 °C among the species (Additional file 1: Supplementary Figure 5). We found the crawling speed was reduced at 40 °C in all the species (Additional file 1: Supplementary Figure 6), which might be a common adaptation of the *Drosophila* larval locomotion at the extreme ambient temperature and/or due to an abnormal physiological reaction at the semi-noxious temperature. The similar tendency, the increase in the bend probability and the decrease in the crawling speed at 40 °C, was also observed when compared with the kinematics at 24 °C (Fig. 10C, D). Intriguingly, in some species, backward crawling, which seldom occurs at 32 °C, could be observed at 40 °C (Fig. 10E). Consequently, these observations showed that at the extreme ambient temperature of 40 °C, the relation between the kinematics indices and habitat temperature did not hold and larvae exhibited common (increase in the crawling speed) and diverse (changes in the bend probability and generation of backward crawling) shifts in the locomotion kinematics among the *Drosophila* species.

### Phylogenetic analyses of interspecies differences in kinematics

Finally, to obtain a hint on how the kinematics parameters diversified across the species developed during the evolutionary history of the genus *Drosophila*, we conducted Bayesian phylogenetic analyses using RevBayes v. 1.0.7 [29, 49]. At first, we inferred phylogenetic trees of the 11 *Drosophila* species based on eight nuclear genes used previously [50] (see the “Methods” section for details). Then, by using our kinematics dataset, we estimated the following three factors in the phylogenetic tree by Bayesian inference: the rate of evolution at each branch of the phylogenetic trees, the relative rates of evolution among the kinematic parameters, and the correlation between the kinematic parameter evolutions [51]. To perform the inference, we constructed a data matrix of eight parameters (the bend probability at 24, 32, and 40 °C; the crawling speed at 24, 32, and 40 °C; backward crawling probability at 40 °C, and the body length), for each of the 11 *Drosophila* species. We assumed that these parameters evolved under a multivariate Brownian-motion model [51–53] (see the “Methods” section for details). We estimated the three factors (the evolution rates at phylogenetic trees, the relative evolution rates among the kinematic parameters, and the correlation between the parameter evolutions) by running a Markov chain Monte Carlo simulation.

**Fig. 11:**
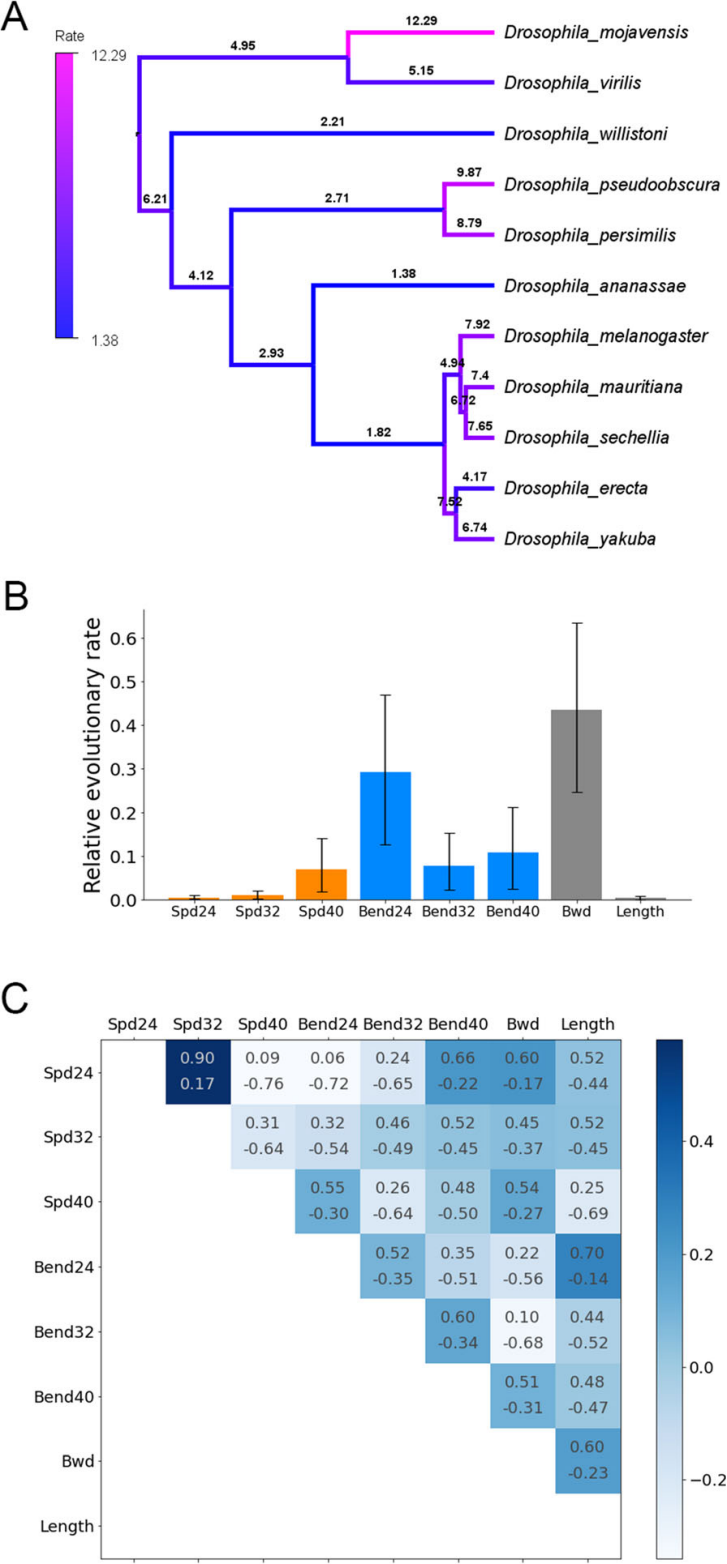
Phylogenetic analyses of larval locomotion in the genus *Drosophila*. **A** Phylogenetic tree estimated from Bayesian inference. The topology was inferred by coding sequences of eight genes of the 11 *Drosophila* species. As a prior distribution for the Bayesian inference, an uncorrelated exponential (UCED) relaxed-clock model was used. The colour and the value of each branch denoted the relative rate of the evolution of the eight kinematic traits. **B** Relative evolution rates of the eight kinematics traits estimated by the Bayesian inference. The mean ± 95% highest posterior density was shown. **C** Correlation between the evolution of eight traits. Numbers represented the range of the 95% highest posterior density. Abbreviations in **B** and **C** were as follows: Spd24, crawling speed at 24 °C; Spd32, crawling speed at 32 °C; Spd40, crawling speed at 40 °C; Bend24, bend probability at 24 °C; Bend32, bend probability at 32 °C; Bend40, bend probability at 40 °C; Bwd, probability of backward crawling; Length, axial body length of larvae

The phylogenetic analyses indicated that the rates of evolution in the kinematics were highly diverse over branches in the phylogenetic tree (Fig. 11A and Additional file 1: Supplementary Figure 7). In addition, the evolutionary rates were distinct among the eight kinematics parameters (Fig. 11B). The bend probability at 24 °C and the probability of backward crawling at 40 °C had a relatively high evolution rate, which might reflect the large diversification of these two parameters among the species (Figs. 9A and 10E).

Some kinematics traits might have evolved cooperatively. To test this possibility, we calculated the correlation of the evolutionary changes between the kinematic parameters. In the correlation analyses of all pairs of the parameters, the crawling speed at 24 °C and the crawling speed at 32 °C were the most correlated (Fig. 11C). On the other hand, the evolutions of the bend probability between at 24 and 32 °C were less correlated. This observation was consistent with the notion that while changes in the bend probability from 24 to 32 °C among the species were diverse (Fig. 7C), all the species showed an increase in the crawling speed by the temperature shift (Fig. 8C). Consequently, the phylogenetic analyses implied that the kinematics indices of larval locomotion evolved differently in distinct branches of a phylogenetic tree with keeping a correlation between specific locomotion traits such as crawling speed at distinct temperatures.

## Discussion

**Fig. 12:**
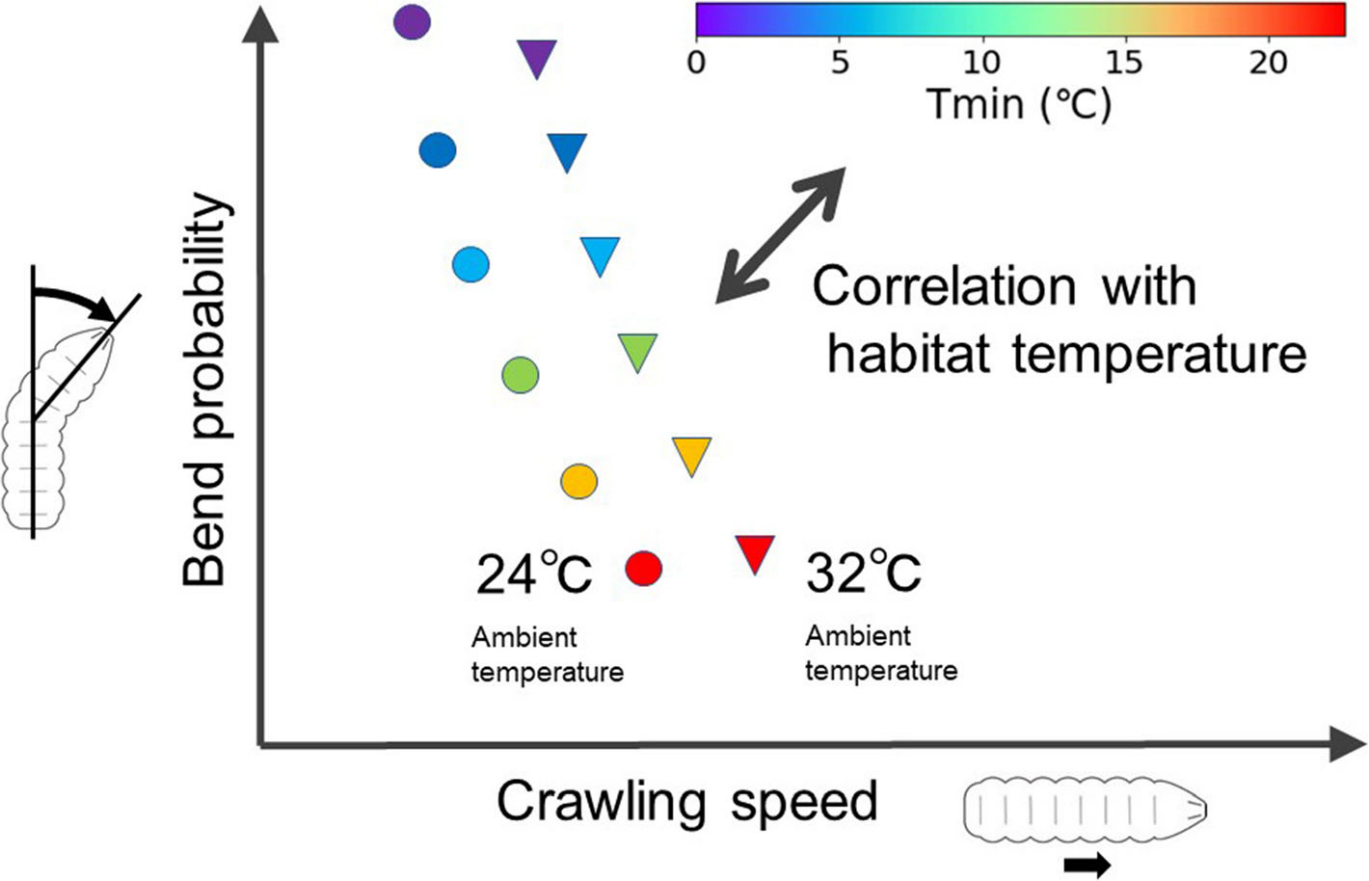
Summary of this study. Disks and triangles denoted the kinematics of each species at 24 °C and 32 °C, respectively

In this work, we investigated the interspecies differences in larval locomotion in the genus *Drosophila*. We used bend probability and crawling speed as measures to examine larval locomotion. Despite the similar appearance of larval bodies in different species, the kinematics of larval locomotion is diverged (Fig. 2). The body length is not a leading factor for the diversity of kinematics (Additional file 1: Supplementary Figure 2). The phylogenetic relationship is also not a major determinant for the kinematics (Figs. 3E and 11A). Considering a previous study showing that phylogenetic relationship does not correlate with the divergence in the morphology of larval neuromuscular junctions [44], genetic drift with random accumulation of neutral mutations is unlikely to underlie the divergence in the larval crawling patterns. In contrast, habitat temperature correlates with both the bend probability and crawling speed at both 24 °C and 32 °C ambient temperature (Fig. 12), which implies the kinematics indices are adapted to ambient temperature in evolution. Phylogenetic analysis by Bayesian inference suggests that the rates of evolution are divergent among the branches of the phylogenetic tree (Fig. 11A). Among the kinematic parameters, the bend probability at the ambient temperature of 24 °C and the backward crawling probability at 40 °C have relatively higher evolution rates than the others, and the evolution of the crawling speed at 24 °C and 32 °C is correlated (Fig. 11B, C). Regarding the questions raised in the “Background” section, our results suggest the following: (1) locomotion kinematics of larvae is divergent among *Drosophila* species, and (2) the habitat temperature is more related to the kinematics indices (the bend probability and crawling speed) than the body length (Fig. 12).

To conduct the quantitative analyses, we measured the larval crawling behaviour in simple experimental conditions: at the constant temperatures and humidity levels and on a flat surface of agarose gel. However, the environmental conditions in the wild for *Drosophila* larvae are far more complicated and diverse [8]. For example, *Dmoj* breeds in cacti in the desert while *Dvir* inhabits in the slime fluxes in temperate and subarctic climates (Additional file 1: Supplementary Figure 3 [8];). Our data in this paper imply that the variation of the kinematics should be related to the diversity of environmental conditions including habitat temperature. Comprehensive and quantitative analyses of larval locomotion in nature would shed light on the causes and meanings of the changes in larval kinematics that appeared in the evolution.

The correlation between habitat temperature and the kinematics raises a question about the underlying mechanisms of adaptive change in kinematics to the habitat temperature. We presume that the adaptive change was likely to be driven by two factors: the pupation positioning and competition over food. Regarding the pupation behaviour, a previous study showed a correlation between path lengths of crawling and heights of pupation location in *D. melanogaster* [16]. Since pupae are immobile, adequate selection of pupation position is vital for their survival. Moisture is one of the key factors that affects the selection of pupation site, since dry environments can cause pupal desiccation, while soaked media might lead to the drowning of pupae [16, 54]. It is reasonable to assume that habitat temperature is a leading factor in determining the moisture levels of microenvironments of *Drosophila* larvae. Accordingly, divergent temperatures in the habitats of flies lead to variability in moisture levels, the ambient moisture affects the pupation position [54], and the pupation position is related to the path length of crawling [16]. These three links might underlie the relationship between the larval locomotion kinematics and habitat temperature of each species.

The second possible mechanism underlying the relation between habitat temperature and locomotion kinematics is related to competition over food. It is suggested that larval feeding is competitive between individuals [55]. There is a positive relation between fitness and density (called Allee effects) in *D. melanogaster* larvae [56], and so female flies aggregate eggs when laying them [55]. Consequently, the larval population tends to be overcrowded [57], and larvae compete over the limited resources to meet their need to consume sufficient amounts of nutrients within limited larval periods [58]. If the available food is limited and larval density is high, larvae need to crawl further [17], which can be an evolutionary driving force to increase the crawling distance. In contrast, if there is plenty of food in the habitat, the crawling distance remains unchanged or even decreases during evolution, because crawling behaviour is energetically costly [14]. Consistent with this implication, the diversification in the larval kinematics in the 11 *Drosophila* species gives rise to the variation in the crawling distance (Fig. 5). Accordingly, feeding conditions, especially the choice of which foods to eat, can affect the kinematics of larval locomotion in evolutionary processes so that the divergence of food to eat can lead to the divergence in locomotion kinematics. In regard to this point, food for larvae of the genus *Drosophila* is divergent and related to their living environments [8–10]. So, the nutritional conditions (nutrient balances, fermentation, etc.) and physical properties (hardness, wetness, etc.) of food to eat vary among the *Drosophila* species, which can drive evolutionary divergence in the kinematics of larval locomotion. Then, the growth of plants, including fruits and vegetables, for larvae is strongly affected by habitat temperature. Therefore, divergence in habitat temperature may affect the locomotion kinematics during evolution through divergence in the foods that larvae feed on, and divergence in requisite feeding behaviour for larval growth. Comprehensive kinematics studies of other *Drosophila* species and quantitative analyses of microenvironments of wild larvae in nature will give us insights into the relationships between ambient temperature and diverse larval locomotion kinematics.

How can we approach the neural circuit mechanisms underlying the interspecies divergence in larval locomotion? Circuit mechanisms in larval locomotion have been examined intensively in *Drosophila melanogaster*. Recent connectomics studies have identified several key neurons for larval locomotion in *Drosophila melanogaster* [20, 30, 59–67]. Regarding bending, the thoracic neuromere was shown to be important in bending in chemotaxis [30]. Especially, the commissural connection is crucial for bending [68]. In addition, a signal from the chordotonal sensory organ is also required to generate bending [69]. Regarding the crawling speed, a group of inhibitory premotor neurons was identified to be critical [64]. Proprioceptive feedback and neuromodulation are both important for the normal crawling speed [12, 63]. Furthermore, the kinematics of the larval locomotion have been measured and investigated quantitatively in detail [26, 31, 33, 34, 70–73] and analysed by mathematical modelling [74–78]. Regarding temperature sensing, neuronal and molecular mechanisms on temperature-guided behaviour have been clarified [22–24, 27, 79–83]. These extensive findings on the cellular and molecular mechanisms on larval crawling in *Drosophila melanogaster* will be an ideal starting point to investigate the evolution of larval behaviour in the genus *Drosophila*. For example, differences in commissure fibre tracts in the central nervous system [68] among the species might underlie the divergence in the bend probability. Interspecies comparison of a group of interneurons PMSIs (period-positive median segmental interneurons), which are involved in the crawling speed [64], would reveal the neural mechanisms on the evolutionary diversification in the crawling speed. Comparative analyses of neural network architectures and gene expression among the *Drosophila* species will relate the evolution of the nervous system to the adaptive diversification in larval behaviour.

## Conclusions

Here, our work suggests interspecific diversity of the larval locomotion kinematics among species in the genus *Drosophila*. The variation is not correlated to the body length but rather to the habitat temperature of the species: larvae of species inhabiting moderate-temperature environments exhibited low bend probability and fast crawling, or long crawling distance, whereas those inhabiting low-temperature environments showed frequent bending and slow crawling, or short crawling distance. Phylogenetic analyses based on Bayesian inference indicate that the evolutionary rate of the kinematic properties is diverse among phylogenetic tree branches. These results suggest that the kinematics of larval locomotion in the genus *Drosophila* diverged under the effects of the ambient temperature of their habitats.

## Methods

### *Drosophila* strains

We used the following fly stocks (17 strains from 11 species): *Drosophila ananassae* (k-s01), *Drosophila erecta* (k-s02), *Drosophila yakuba* (k-s03), *Drosophila melanogaster* (k-s04), *Drosophila sechellia* (k-s10), *Drosophila persimilis* (k-s11), *Drosophila pseudoobscura* (k-s12), *Drosophila willistoni* (k-s13), *Drosophila virilis* (k-s14), and *Drosophila mojavensis* (k-s15), *Drosophila melanogaster* collected at Kyoto (k-aba029) and Iriomote (k-aba032), *Drosophila ananassae* collected at Ogasawara (k-aaa027) and Iriomote (k-aaa309), and *Drosophila virilis* collected at Hokkaido (E-15601) and Toyama (E-15605) from KYORIN-Fly, Fly Stocks of Kyorin University and *Drosophila mauritiana* (#900020) from KYOTO Stock Center (DGRC) at the Kyoto Institute of Technology. These strains have been maintained at 23 °C (except for *Drosophila persimilis* at 20 °C) in the stock centres for more than 10 years. All animals were raised on standard cornmeal-based food at 25 °C in the authors’ laboratory before the experiments for less than 3 months after the acquisition from the stock centres.

### Recording larval locomotion

Third-instar wandering larvae of each strain were picked up and gently washed in deionised water. Residual food on the larvae was brushed off with a paintbrush. An agarose stage (size 9 cm × 9 cm × 5 mm; 1.5% agarose; RIKAKEN STAR agarose powder #RSV-AGRP-100G) was placed on a temperature-controlled plate (Cool Plate, AS ONE Corporation, Japan), and the temperature of the surface was kept at 24 ± 1 °C, 32 ± 1 °C, or 40 ± 1 °C. Eight to ten larvae were placed on the agarose arena with a paintbrush. The larvae were illuminated with infrared light, which larvae cannot see (LDQ-150IR2-850, wavelength = 850 nm, CCS Inc., Japan) and recorded at five frames/s with a CCD camera (CGE-B013-U, MIGHTEX, Canada; resolution 1280 pixels × 960 pixels) and an infrared filter (775LP filter, Omega Optical, USA), and saved as a series of bitmap files. In our setting, the scale of the image is 0.13 mm/pixel. For each of the 17 strains (from 11 species) and at each of the three ambient temperatures, we repeated the measurement three times.

### Larva tracking

We obtained a time series of bitmap image files by the procedure described above. The bitmap files were converted to tiff format by Fiji (https://imagej.net/fiji). In cases where larvae accumulated at the first frame, we removed the first several images until the larvae dispersed enough to be identified individually. The *x* and *y* coordinates of the centroid and the bend angle (Fig. 1E) of individual larvae were obtained by using the FIMTrack software [28]. We used tracking data of single larvae that were continuously tracked for more than 300 frames (which corresponds to 1 min). For the intraspecific analysis in Fig. 4, single larvae with more than 150 continuous frames were tracked. In case a larva collided with another larva in the middle of the recording and the larva was traced differently before and after the collision with distinct labels, we treated the two traces as two larvae. The crawling speed of each larva was obtained as the median of crawling speed in the whole trace of the larva. Bend probability of each larva was calculated as the ratio of the number of frames showing bending (Fig. 3 for the definition of bending) to the total number of the frames of its trace. The data of the three movies for each species/strain were merged. The coordinates and bend angles were smoothed and plotted by Python 3.7. For the visualisation of the traces in Fig. 1E, F, we smoothed the data by a uniform window function with five frames. The number of pixels in a single larva ranges from about 150 (*Dyak*) to 500 (*Dpse*) that is sufficient to capture the body bend angles.

### Clustering analysis of kinematics

For clustering analysis of the plots in Fig. 2A, we made a probability distribution. We discretised the centroid speed axis by an interval of 0.1 mm/s between 0 and 4 mm/s and the bend angle axis by an interval of two degrees between 0 and 140°, then created 2800 bins in total (40 bins in the speed axis and 70 bins in the bend angle axis). For each scatter plot, we counted the number of points in each bin and obtained probability density by normalisation with the total number of points. We calculated the Kullback-Leibler divergence (KL) of probability distributions of two species *p*(bin) and *q*(bin) as follows:

$$ \mathrm{KL}\left(p,q\right)={\sum}_{\mathrm{bin}=1}^{2800}p\left(\mathrm{bin}\right)\ast \log \frac{p\left(\mathrm{bin}\right)}{q\left(\mathrm{bin}\right)}. $$

Since KL(*p*,*q*) is an asymmetric measure (KL(*p*, *q*) ≠ KL(*q*, *p*)), we calculated the Jensen-Shannon divergence (JS), which is a symmetric alternative to the Kullback-Leibler divergence:

$$ \mathrm{JS}\left(p,q\right)=\frac{1}{2}\ \left\{\mathrm{KL}\left(p,q\right)+\mathrm{KL}\left(q,p\right)\right\}=\mathrm{JS}\left(q,p\right). $$

Based on the Jensen-Shannon divergence, we conducted the hierarchical clustering using Python 3.7 and the SciPy library.

### Habitat temperature of the *Drosophila* species

Global climate data were obtained from the WorldClim data website (https://www.worldclim.org/data/worldclim21.html). We used minimum, mean, and maximum temperature in the world at a spatial resolution of 5 min (9.25 km × 9.25 km). Habitat regions of each *Drosophila* species were obtained from the literature [7, 41, 42] and the DrosWLD-Species database (https://bioinfo.museum.hokudai.ac.jp/db/modules/stdb/index.php?action=tbl&tbl_id=21) and traced them on the world map (2160 pixels × 4320 pixels) obtained from the WorldClim by using Adobe Illustrator software (Adobe Inc., USA). The maps in Additional file 1: Supplementary Figure 2 were generated by Adobe Illustrator, and the statistics of the temperature within each species’ habitat were calculated by Python 3.7.

### Statistical analysis

Statistical calculation was conducted by Python 3.7 in the following analyses: Kruskal-Wallis test in Fig. 3C, D, Pearson correlation in Figs. 6B, C; 7B, C; 8B, C; and Additional file 1: Figure 2B, 2C, 4B and 5B, and Mann-Whitney *U* test in Fig. 7A and 8A and Additional file 1: Supplementary Figure 5A and 6A.

### Phylogenetic analyses

We adopted Bayesian statistics for phylogenetic analyses. In all the phylogenetic analyses, we used the software package RevBayes v.1.0.7 [29].

We estimated the *Drosophila* phylogenetic trees (Fig. 8A) by using eight nuclear loci from Turelli et al. [50]: *aldolase*, *bicoid*, *enolase*, *esc*, *transaldolase*, *white*, *wingless*, and *yellow*. We used *Scaptodrosophila lebanonensis* (*S. lebanonensis*) as an outgroup. Coding sequences of these genes for all the species except for *D. mauritiana* and *S. lebanonensis* were obtained from Kalay et al. [51]. Coding sequences of the genes of *D. mauritiana* and *S. lebanonensis* were obtained from the NCBI website (https://www.ncbi.nlm.nih.gov/). The genes were aligned with MAFFT version 7 [84]. We selected the eight genes out of twenty genes described in Turelli et al. [50] because homologous genes of the other twelve genes were not identified in the genome assembly of *D. mauritiana*. Based on the eight coding sequences of the twelve species, we performed chronogram analyses described in Turelli et al. [50] and obtained the phylogenetic tree in Fig. 8A, where the root is the branch of *S. lebanonensis* and the length of each branch indicates the relative time in the evolution. We repeated the phylogeny inference four times to obtain four trees to check the robustness of this inference in the following analysis.

Based on the phylogenetic tree obtained above, we analysed the evolution of kinematics parameters. We used eight parameters (bend probability at 24, 32, and 40 °C; crawling speed at 24, 32, and 40 °C; backward crawling probability at 40 °C; and body length), for each of the 11 *Drosophila* species. In this analysis, while the topology of the phylogenetic tree is unchanged, the rates of evolution of the kinematic parameters and the rates of evolution of the branches of the phylogenetic tree were estimated by Bayesian inference. We assumed the kinematics parameters evolve under a multivariate Brownian-motion model [52, 53]. This model consists of two components: the relative rates of evolution among the kinematics parameters and the correlation between each pair of the kinematics parameters [51]. The Brownian model assumes that the changes in the kinematics parameters during evolution are additive, which has two consequences: (1) parameters can become negative, and (2) evolution rates can depend on the scale of the kinematics parameters. To cope with these properties, we adopted log-transformation to the kinematics parameters as described previously [51]. This transformation guarantees that the original kinematics parameters (which are exponentials of the log-transformation) remain positive and controls the size issue by transforming additive changes to multiplicative ones [51]. For prior models for the evolution rates of the branches, we specified an uncorrelated exponential (UCED) relaxed-clock model, where the rate of each branch is drawn independently from an exponential distribution. We ran the Markov chain Monte Carlo (MCMC) simulation of UCED with each of the four phylogenetic trees described in the previous paragraph (Fig. 11). Consistent results were obtained by another prior model, an uncorrelated gamma (UCG) relaxed-clock model (Supplementary Figure 7), which indicates the predictions are robust to the choice of priors.

## Supplementary Information

**Additional file 1: Figure S1.** The negative correlation between the crawling speed and bend probability is robust to the angle threshold for the definition of bend. (A-C) Scatter plots of the bend probability at 24°C against the crawling speed at 24°C. (A) The angle threshold for bend was 10 degrees. (B) The threshold was 20 degrees. (C) The threshold was 30 degrees. Median ± sem was shown. B was the same panel as Figure 3C represented here for comparison. (D) Scatter plot of the median of absolute bend angle at 24°C against the crawling speed at 24°C. The red lines showed the linear regression functions and the shaded areas represented the 95% confidence band. The point estimates of the Pearson correlation and its 95% confidence intervals were − 0.80 and [-0.95, -0.39] in A, -0.76 and [-0.94, -0.30] in B, -0.71 and [-0.92, -0.19] in C, and -0.66 and [-0.90, -0.11] in D. **Figure S2.** Interspecies comparison between larva length and kinematics parameters. (A) Axial body length of larvae of the 11 species. Sample numbers were the following: Dvir: n=69; Dmel: n=72; Dmoj: n=82; Dper: n=76; Dpse: n=60; Dsec: n=68; Dwil: n=66; Dere: n=69; Dmau: n=69; Dana: n=68; Dyak: n=56. (B) Scatter plot of the bend probability at 24°C against the larva length of the 11 species. (C) Scatter plot of the crawling speed at 24°C against the larva length of the 11 species. In B and C, median ± sem was shown. The red lines showed the linear regression functions and the shaded areas represented the 95% confidence band. The point estimates of the Pearson correlation and its 95% confidence intervals were 0.55 and [-0.07, 0.87] in B and 0.04 and [-0.57, 0.63] in C. **Figure S3.** World maps of histograms of habitat temperature of the Drosophila species. The top of each panel showed a map of the habitat of the labelled species. The bottom on each panel showed a histogram of minimum (green) and maximum (magenta) temperatures within the habitat. Asterisks denoted the warmest peak in the minimum habitat temperature. The temperature at the peak was called Tmin in this study. **Figure S4.** Relationship between the kinematics of larval locomotion and the range of habitat temperature of the Drosophila species (A) Scatter plot of the bend probability speed at 24°C against the range of habitat temperature of the 11 species. (B) Scatter plot of the crawling speed probability at 24°C against the larva length of the 11 species. Median ± sem was shown. The red lines showed the linear regression functions and the shaded areas represented the 95% confidence band. The point estimates of the Pearson correlation and its 95% confidence intervals were 0.65 and [0.08, 0.90] in A and -0.69 and [-0.91, -0.15] in B. **Figure S5.** Bend probability in the 11 Drosophila species at 40°C. (A) Bend probability of the 11 species at 32°C and 40°C. The data at 32°C were the same as in Figure 4A. (B) Scatter plot of the bend probability at 40°C against at 32°C. Sample numbers at 40°C were the following: Dvir: n=20; Dmel: n=28; Dmoj: n=23; Dper: n=21; Dpse: n=16; Dsec: n=27; Dwil: n=27; Dere: n=22; Dmau: n=26; Dana: n=23; Dyak: n=20. Sample numbers at 32°C were described in the legend of Figure 4. Median ± sem was shown in B. **Figure S6.** Crawling speed in the 11 Drosophila species at 40°C. (A) Crawling speed of the 11 species at 32°C and 40°C. The data at 32°C were the same as in Figure 5A. (B) Scatter plot of the crawling speed at 40°C against at 32°C. Sample numbers at 40°C were the following: Dvir: n=20; Dmel: n=28; Dmoj: n=23; Dper: n=21; Dpse: n=16; Dsec: n=27; Dwil: n=27; Dere: n=22; Dmau: n=26; Dana: n=23; Dyak: n=20. Sample numbers at 32°C were described in the legend of Figure 5. Median ± sem was shown in B. **Figure S7.** Phylogenetic analyses of larval locomotion based on distinct prior models. (A) Phylogenetic tree estimated from Bayesian inference with an uncorrelated exponential (UCED) relaxed-clock model as the prior. Values of each branch denoted the range of 95% highest posterior density of the inference of the evolution rates shown in Figure 11A. (B and C) Phylogenetic tree estimated from Bayesian inference with an uncorrelated gamma (UCG) relaxed clock model. Values of each branch showed the evolution rate (B) and its range of 95% highest posterior density (C).

## Data Availability

All data generated or analysed during this study are included in this article, its supplementary information file, or in the figshare repository: 10.6084/m9.figshare.15041829 [85].
